# The role of CSF1R-dependent macrophages in control of the intestinal stem-cell niche

**DOI:** 10.1038/s41467-018-03638-6

**Published:** 2018-03-28

**Authors:** Anuj Sehgal, David S. Donaldson, Clare Pridans, Kristin A. Sauter, David A. Hume, Neil A. Mabbott

**Affiliations:** 1 The Roslin Institute & Royal (Dick) School of Veterinary Sciences, University of Edinburgh, Easter Bush, Midlothian, EH25 9RG UK; 2 MRC Centre for Inflammation Research, University of Edinburgh, The Queen’s Medical Research Institute, 47 Little France Crescent, Edinburgh, EH16 4TJ UK; 3 Mater Research-University of Queensland, Translational Research Institute, Woolloongabba, QL 4102 Australia

## Abstract

Colony-stimulating factor 1 (CSF1) controls the growth and differentiation of macrophages.CSF1R signaling has been implicated in the maintenance of the intestinal stem cell niche and differentiation of Paneth cells, but evidence of expression of CSF1R within the crypt is equivocal. Here we show that CSF1R-dependent macrophages influence intestinal epithelial differentiation and homeostasis. In the intestinal lamina propria CSF1R mRNA expression is restricted to macrophages which are intimately associated with the crypt epithelium, and is undetectable in Paneth cells. Macrophage ablation following CSF1R blockade affects Paneth cell differentiation and leads to a reduction of *Lgr5*^+^ intestinal stem cells. The disturbances to the crypt caused by macrophage depletion adversely affect the subsequent differentiation of intestinal epithelial cell lineages. Goblet cell density is enhanced, whereas the development of M cells in Peyer’s patches is impeded. We suggest that modification of the phenotype or abundance of macrophages in the gut wall alters the development of the intestinal epithelium and the ability to sample gut antigens.

## Abstract (web summary)

Colony stimulating factor 1 controls the growth and differentiation of macrophages. Here the authors demonstrate that its blockade depletes gut macrophages and indirectly affects gut epithelial cell differentiation as the macrophages help maintain the Paneth and stem cells in intestinal crypts.

## Introduction

Mucosal surfaces such as those in the gastrointestinal and respiratory tracts are continuously exposed to commensal and pathogenic microorganisms. In the intestine a single layer of epithelial cells bound by tight-junctions limits the access of these microorganisms to the underlying host tissues. This epithelium is self-renewing and is continually replaced every 5–7 days. The crypts at the base of each intestinal villus contain populations of cycling leucine-rich repeat-containing G protein-coupled receptor 5 (Lgr5)-expressing intestinal stem cells^1^. These stem cells produce highly proliferative transit-amplifying daughter cells that, depending on the stimuli they receive, can differentiate into all the epithelial cell populations within the lining of the small intestine, including: enterocytes, goblet cells, enteroendocrine cells, tuft cells, and Paneth cells. Differentiated cell types, with the exception of the Paneth cells, migrate along the epithelium of the villus where they perform their physiological function before being subsequently lost via apoptosis as they reach the villus tip. Paneth cells remain located within the base of the crypts where they are interspersed between the Lgr5^+^ intestinal stem cells. Paneth cells release antimicrobial products such as lysozyme and alpha defensins which help to protect the crypt from bacterial infection, alongside homeostatic factors such as Wnt-signaling molecules which are essential for the maintenance of the Lgr5^+^ intestinal stem cells. Accordingly, when Paneth cells are specifically depleted there is a concomitant reduction in Lgr5^+^ stem cells^2^.

The lamina propria of the intestine contains an abundant population of macrophages that are renewed continuously from the circulating monocyte population^3,4^. Monocyte-macrophage differentiation is controlled by colony-stimulating factor (CSF1), and mice lacking this factor (*op/op*) or the receptor, CSF1R, are profoundly macrophage deficient^5^. CSF1 signaling is also required for maintenance of the macrophage populations in adult mice. Treatment of adult mice with a blocking anti-CSF1R antibody produces an almost complete depletion of tissue macrophage populations in most organs, including the intestinal lamina propria^6^. Conversely, treatment of mice with CSF1^7^, or with a recently-developed recombinant CSF1-Fc conjugate, induces extensive macrophage infiltration into tissues^8^.

The *op*/*op* mouse also lacks lysozyme-expressing Paneth cells, and shows a commensurate reduction in *Lgr5* expression and cell proliferation in the crypt^9,10^. Immunostaining with an anti-CSF1R antiserum suggested that the protein was expressed by Paneth cells implying that CSF1 directly regulates their development^9–11^. By contrast, a *Csf1r*-EGFP reporter mouse, in which the *Csf1r* promoter drives the expression of EGFP^12^, labels tissue macrophages but not the Paneth cells, or indeed any epithelial cell lineage throughout the lining of the small intestine^13^.

CSF1-dependent macrophages exhibit many important roles in the maintenance of tissue homeostasis and repair^14^. For example, the macrophages in the muscularis externa of the wall of the gut can respond to luminal bacterial infections, produce bone morphogenetic protein 2 and interact with enteric neurons to regulate gastrointestinal motility^15,16^. The neurons in turn, produce CSF1. Therefore, in the current study, we tested the hypothesis that the effect of CSF1R blockade on the maintenance of Paneth cells in the intestinal crypts was indirect. Indeed, we show here that CSF1R-dependent macrophages are essential for the constitutive homeostatic maintenance of the intestinal crypt.

In gut-associated lymphoid tissues (GALT), Lgr5^+^ intestinal stem cells within the dome-associated crypts also give rise to M cells^17^. These unique epithelial cells are specialized for the transcytosis of lumenal particulate antigens and pathogens across the follicle-associated epithelium (FAE)^18^. The transcytosis of particulate antigens from the gut lumen by M cells is an important first step in the induction of an efficient mucosal immune response^19–21^. Since Lgr5^+^ intestinal stem cells are adversely affected in absence of Paneth cells^2^ or CSF1R signaling^9,10^, we also tested the hypothesis that prolonged CSF1R blockade indirectly affects the functional differentiation of M cells. A link between macrophage function and antigen sampling provides an obvious mechanism to ensure that antigens derived from the gut are recognized by the innate immune system.

In this study, we show that CSF1R mRNA expression is undetectable in Paneth cells within intestinal crypts and is instead restricted to macrophages which are intimately associated with the crypt epithelium. The depletion of these macrophages following prolonged CSF1R blockade disturbs intestinal crypt homeostasis, affecting the differentiation of Paneth cells and Lgr5^+^ intestinal stem cells. The disturbances to the crypt caused by macrophage depletion adversely affect the subsequent differentiation of intestinal epithelial cell lineages, changing the balance between goblet cell and M-cell differentiation. Taken together, our observations reveal that CSF1R-dependent crypt-associated macrophages are constitutively required to maintain the intestinal stem-cell niche in the small intestine. This suggests that modification of the phenotype or abundance of macrophages in the gut wall, for example after pathogen infection, could adversely affect the development of the intestinal epithelium and the ability of the mucosal immune system to sample particulate antigens from the gut lumen.

## Results

### Prolonged CSF1R blockade depletes macrophages throughout the gut

**Fig. 1:**
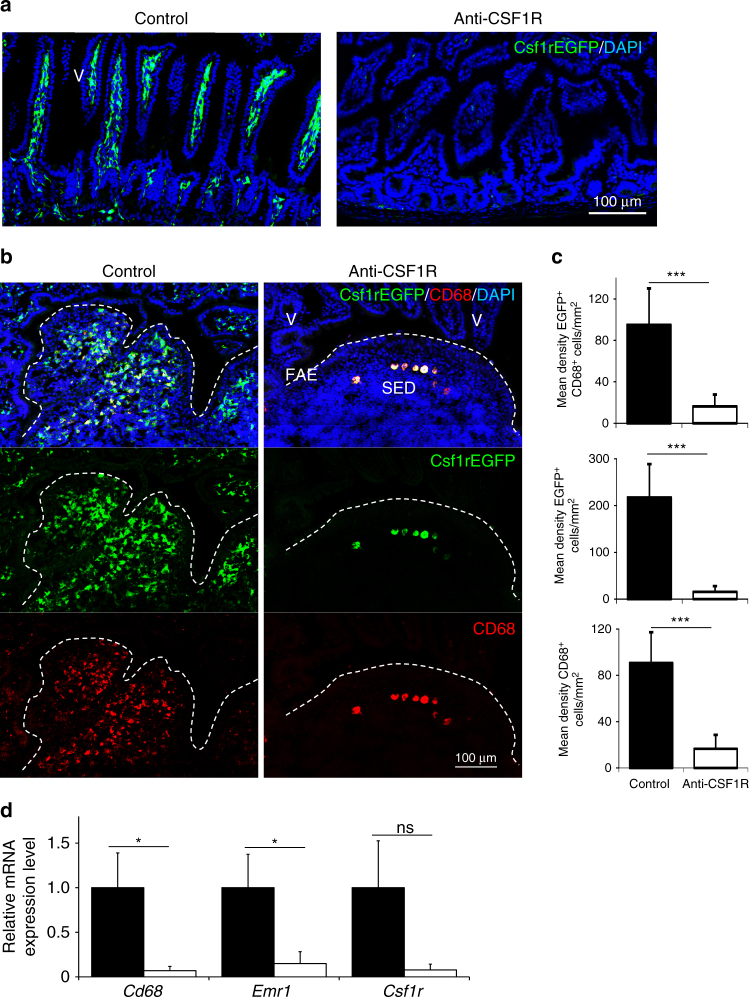
Prolonged anti-CSF1R blockade depletes macrophages in the gut. *Csf1r*-EGFP mice were treated with anti-CSF1R mAb or control-IgG (control) for 6 weeks before analysis. **a** Depletion of *Csf1r*-EGFP^+^ (green) macrophages in the gut wall of anti-CSF1R mAb-treated mice. Scale bar, 100 µm. Images are representative of four mice/group from three independent experiments. **b** Detection of *Csf1r*-EGFP^+^ (green) and CD68^+^ (red) macrophages in Peyer’s patches by IHC. FAE, follicle-associated epithelium; SED, subepithelial dome; V, villus. Broken lines indicate the FAE boundary. Scale bar, 100 µm. **c** Morphometric analysis of the density of *Csf1r*-EGFP^+^CD68^+^ macrophages in Peyer’s patches. Data represent mean ± SD from 2 to 10 sections from 3 to 4 mice/group. ***P < 0.001, two-tailed unpaired Student’s *t*-test. **d** RT–qPCR analysis of *Cd68*, *Emr1*, and *Csf1r* expression in Peyer’s patches. Bars represent mean ± SEM. Data are derived from 3 to 4 mice/group. *P < 0.05, two-tailed unpaired Student’s *t*-test. NS not significant

Prolonged CSF1R blockade was achieved by treatment of C57BL/6J wild-type mice or *Csf1r*-EGFP mice with anti-CSF1R mAb M279 (or normal rat IgG as a control) three times a week for a 6 week period^13^. The efficacy of the CSF1R blockade was confirmed by the >95% reduction of *Csf1r*-EGFP^+^ macrophages throughout the wall of the gut (Fig. 1a), in Peyer’s patches (Fig. 1b, c) and bone marrow (Supplementary Fig. 1). The expression of *Csf1r* and typical macrophage-specific transcripts including *Cd68* and *Emr1* (also known as *Adgre1*, encoding the F4/80 antigen) was also reduced in mRNA from the ileum of anti-CSF1R-treated mice (Fig. 1d).

### Constitutive CSF1R stimulation maintains Paneth cell maturation

**Fig. 2:**
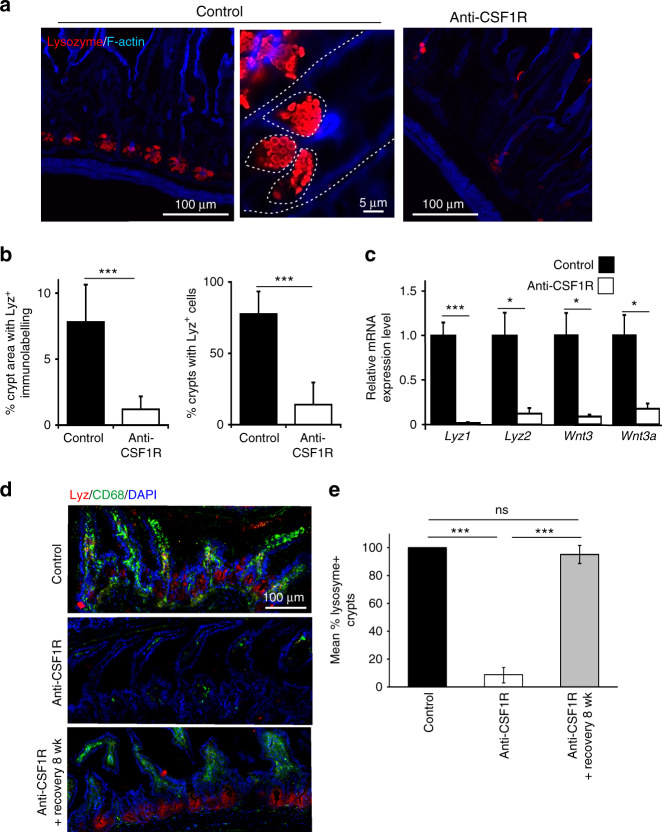
Prolonged anti-CSF1R blockade leads to a loss of lysozyme-expressing Paneth cells in the crypts. Mice were treated with anti-CSF1R mAb or control-IgG (control) for 6 weeks before subsequent analysis. **a** IHC analysis shows loss lysozyme-expressing Paneth cells (red) in intestinal crypts after anti-CSF1R-treatment. Broken line indicates crypt and Paneth cell boundaries. Images are representative of 4 mice/group from 3 independent experiments. **b** Morphometric analysis of the density of lysozyme-expressing Paneth cells in the crypts after anti-CSF1R mAb treatment (open bars) when compared to controls (closed bars). Data represent mean ± SD from 6 to 20 sections from four mice/group, and are representative of data derived from three independent experiments. ***P < 0.001, Mann–Whitney *U*-test. **c** RT–qPCR analysis shows negligible expression of *Lyz1, Lyz2*, *Wnt3*, and *Wnt3a* in mRNA from isolated intestinal crypts following treatment with anti-CSF1R mAb. Data are normalized to the expression of *Gapdh*. Bars represent mean ± SEM. Data are derived from four mice/group. *P < 0.05; ***P < 0.001, two-tailed unpaired Student’s *t*-test. **d** Mice were treated with anti-CSF1R mAb or control-IgG (control) for 6 weeks. A parallel group of mice were allowed to recover for 8 week following anti-CSF1R mAb. IHC analysis shows loss lysozyme-expressing Paneth cells (red) in intestinal crypts and CD68^+^ macrophages (green) in the gut wall after anti-CSF1R-treatment. The presence of these cells was restored within an 8 week recovery period following anti-CSF1R-treatment. Scale bar 100 µm. **e** Morphometric analysis of the % crypts with lysozyme-expressing Paneth cells mice from each treatment group. Data represent mean ± SD from sections from 3 to 4 mice/group, and 30 to 120 crypts/section, and are representative of data from three independent experiments. ***P < 0.001, one-way ANOVA with Tukey’s post hoc test. NS not significant

**Fig. 3:**
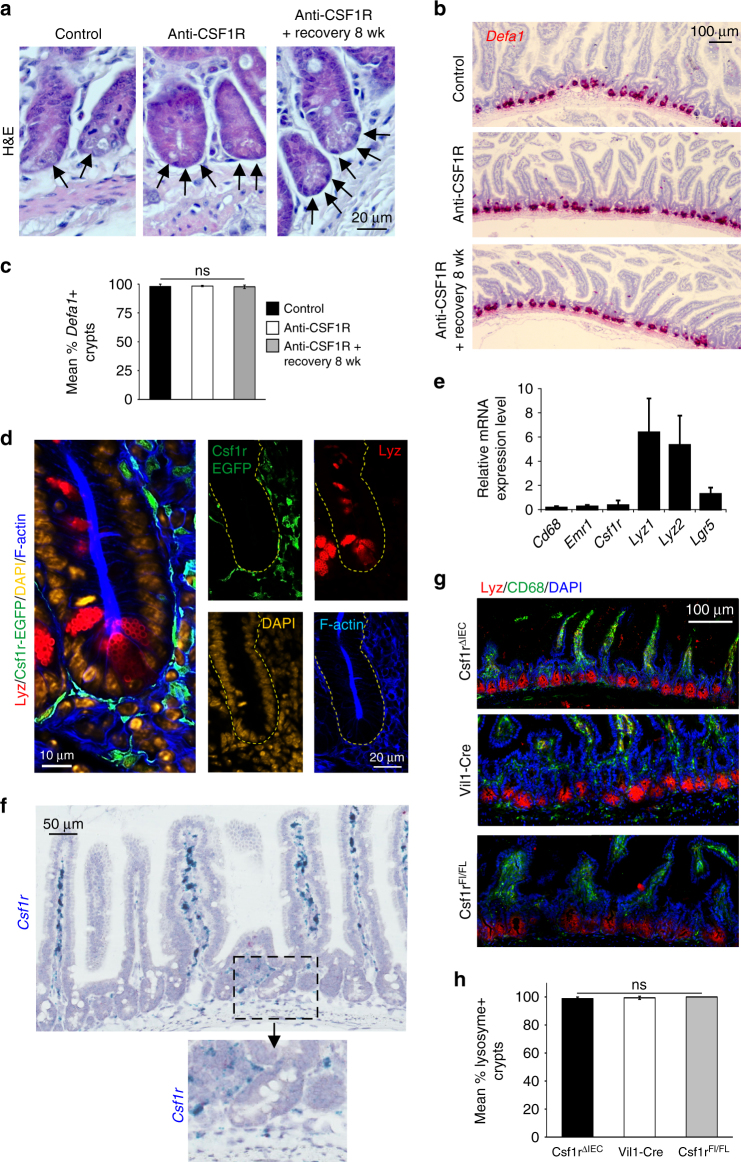
Paneth cells do not express CSF1R mRNA and protein. Mice were treated with anti-CSF1R mAb or control-IgG (control) for 6 weeks before analysis. A parallel group of mice were allowed to recover for 8 wk following anti-CSF1R mAb treatment. **a** Analysis of H&E stained intestines revealed that Paneth cells containing secretory granules (arrows) were present following prolonged CSF1R blockade. Scale bar, 20 µm. Representative images from 3 mice/group are shown. **b** RNA in situ hybridization analyses showed that *Defa1* mRNA (red) was abundantly expressed in the intestinal crypts of mice treated with anti-CSF1R mAb. Scale bar, 100 µm. Representative images from 3 mice/group are shown. **c** Morphometric analysis of the % crypts with *Defa1*-expressing Paneth cells in the crypts of mice from each treatment group. Data represent mean ± SD derived from 3 mice/group, and 96–100 crypts/section. ns, not significant, one-way ANOVA with Tukey’s post hoc test. **d** IHC analysis of the crypts of untreated *Csf1r*-EGFP mice shows Paneth cells do not express CSF1R. In the crypt EGFP expression was only detected in cells with a macrophage morphology. **e** RT–qPCR analysis shows negligible expression of *Cd68*, *Emr1*, and *Csf1r* in mRNA from isolated intestinal crypts. Data are normalized to the expression of *Gapdh*. **f** RNA in situ hybridization analyses showed that *Csf1r* mRNA (blue) was not expressed in the epithelium of the small intestine. Scale bar, 50 µm. Inset shows absence of *Csf1r* mRNA in the epithelium of the intestinal crypt. **g** IHC analysis of lysozyme-expressing Paneth cells (red) in intestinal crypts and CD68^+^ macrophages (green) in the gut wall of Csf1r^ΔIEC^, Vil1-Cre and Csf1r^FL/FL^ mice. Representative images from three mice/group are shown. Scale bar 100 µm. **h** Morphometric analysis revealed that the % crypts with lysoszyme-expressing Paneth cells was similar in the intestines of Csf1r^ΔIEC^, Vil1-Cre and Csf1r^FL/FL^ mice. Data represent mean ± SD derived from three mice/group, and 34–118 crypts/section. NS not significant, one-way ANOVA with Tukey’s post hoc test

Previous studies have shown that the status of the intestinal crypts is disturbed in mice lacking CSF1R signaling^9–11^. The number and density of lysozyme-expressing Paneth cells within the intestinal crypts was significantly reduced after prolonged CSF1R blockade (Fig. 2a, b). A similar reduction in lysozyme mRNA expression, as well as the Paneth cell-derived homeostatic factors *Wnt3* and *Wnt3a* was observed in mRNA from crypts isolated from the intestines of anti-CSF1R mAb-treated mice (Fig. 2c). The effects of CSF1R blockade on Paneth cell status were transient. Lysozyme expression in Paneth cells in intestinal crypts was restored to the same levels as control-treated mice when the mice were allowed to recover for 8 wk following anti-CSF1R mAb treatment (Fig. 2d, e). Prolonged CSF1R blockade did not, in fact, lead to the depletion of Paneth cells. Cells containing abundant cytoplasmic secretory granules clearly remained in the intestinal crypts of mice treated with anti-CSF1R mAb (Fig. 3a). Paneth cells characteristically secrete a large range of antimicrobial factors including alpha defensins. RNA in situ hybridization analyses indicated that *Defa1* (encoding alpha-defensin 1) mRNA was still abundant in the intestinal crypts of mice treated with anti-CSF1R mAb (Fig. 3b). Furthermore, after prolonged CSF1R blockade the number of crypts with *Defa1* mRNA-expressing Paneth cells was similar to those observed in the intestines of control-treated mice (Fig. 3b, c). Taken together, these data clearly indicate that CSF1R signaling is not required for Paneth cell survival, but instead, controls their differentiation.

### Paneth cells do not express CSF1R

The CSF1R dependence of Paneth cells has been attributed to the expression of CSF1R in these cells^9,10^, but could also reflect an indirect requirement for macrophages in intestinal development. For example, CSF1-deficient mice lack insulin-secreting beta cells in the pancreas^22^, but these cells were not depleted in ant-CSF1R treated mice^13^. Using confocal imaging we examined the expression of CSF1R in the intestines of the *Csf1r*-EGFP reporter “MacGreen” mouse in which the *Csf1r* promoter drives the expression of EGFP^12^. In these mice, the only additional sites of EGFP expression are placental trophoblasts, accurately reflecting the expression of *Csf1r* mRNA in these cells from a distal, unique promoter^12^, and granulocytes which express *Csf1r* mRNA but do not translate CSF1R protein^23^. The expression of EGFP in these mice is highly stable and reliably identifies *Csf1r*-expressing cells even in situations where *Csf1r* mRNA is depleted, such as following the trans-differentiation of macrophages into smooth muscle-like cells^24^. In the intestines of *Csf1r*-EGFP mice, EGFP expression was not detected in lysozyme-expressing Paneth cells in the intestinal crypts (Fig. 3d). As anticipated, no background fluorescence was observed in the intestines of non-transgenic C57BL/6 control mice (Supplementary Fig. 2). The absence of CSF1R expression in Paneth cells is further supported by the negligible expression of *Csf1r* in mRNA from isolated intestinal crypts (Fig. 3e), and the absence of *Csf1r* mRNA in data from independent mRNA sequencing studies of intestinal crypts and individual cell populations derived from them and in deep CAGE sequence data from the FANTOM consortium (Supplementary Fig. 3–5)^25–27^. To confirm the lack of expression, we performed RNA in situ hybridization analysis on small intestines from naïve C57BL/6J wild-type mice. *Csf1r* mRNA expression was not detected in epithelial cells within the crypts or anywhere within the gut epithelium (Fig. 3f). To further confirm that *Csf1r* is not expressed functionally in epithelial cells at any stage, we applied a conditional knockout using Villin-Cre, to target *Csf1r*-deficiency to epithelial cells, including Paneth cells: Csf1r^ΔIEC^ mice. The same promoter was used previously to drive tamoxifen-inducible Cre-mediated deletion of *Csf1r*^9^. Vil1-Cre mice and Csf1r^FL/FL^ mice were used as transgenic controls. The numbers of lysozyme-expressing Paneth cells were unchanged in the intestinal crypts of Csf1r^ΔIEC^ mice compared to Vil1-Cre and Csf1r^FL/FL^ transgenic control mice (Fig. 3g, h). Paneth cell morphology and expression of *Defa1* were also unaffected in the intestines of Csf1r^ΔIEC^ mice (Supplementary Fig. 6). These data do not support the proposed direct role for CSF1R signaling in small intestinal crypt homeostasis^9,10^. Instead, an indirect role is supported by the close association between the CSF1R-expressing macrophages and the crypt epithelium.

**Fig. 4:**
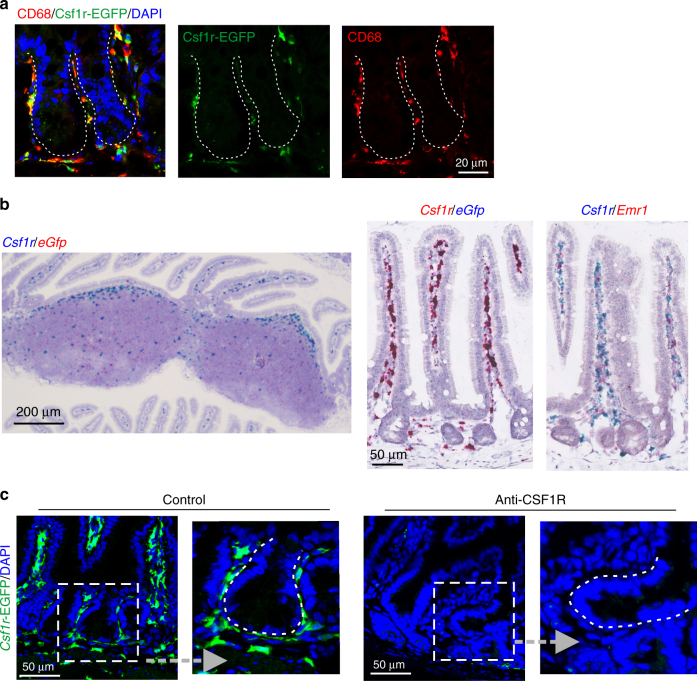
Macrophages are the only cells which express CSF1R within intestinal crypts. **a** IHC analysis shows *Csf1r*-EGFP^+^CD68^+^ macrophages are in direct intimate contact with the crypt epithelial cell layer. Scale bar, 20 µm. **b** Left-hand panel shows the distribution of *Csf1r* (blue) and *eGfp* (red) mRNA expression in Peyer’s patches. Scale bar, 200 µm. In the gut wall *Csf1r* (red, middle panel; blue, right-hand panel) and *eGfp* (blue) mRNA expression was only expressed by macrophages (*Emr1*, red; right-hand panel). Scale bar, 50 µm. **c** IHC analysis shows that in the crypts and gut wall of *Csf1r*-EGFP mice, *Csf1r*-EGFP^+^ macrophages (green) were depleted after CSF1R blockade. Sections in **a**, **c** were counterstained with DAPI to detect cell nuclei (blue). Broken lines indicate the crypt epithelial cell layer boundary. Scale bar, 50 µm. Representative images are shown from 3 to 4 intestinal segments from 4 to 8 mice/group, from three independent experiments

In the vicinity of the intestinal crypt, *Csf1r*-EGFP expression was detectable in CD68^+^ macrophages in direct intimate contact with the epithelial cell layer with frequent extended processes contacting the epithelial cells within it (Figs. 3d and 4a; Supplementary Fig. 7; Supplementary movie 1). Two-color RNA *in situ* hybridization analyses confirmed that the EGFP expression observed in the intestine and Peyer’s patches directly mirrored the cellular distribution of *Csf1r* mRNA expression, and that the *Csf1r*-expressing cells also expressed the macrophage marker *Emr1* (Fig. 4b). Furthermore, in the crypts and gut wall of *Csf1r*-EGFP mice, *Csf1r*-EGFP^+^ macrophages were depleted after CSF1R blockade (Fig. 4c). Consistent with the proposed indirect role of macrophages, as shown in Fig. 2d, e, the restoration of lysozyme expression in Paneth cells coincided with the reappearance of macrophages in the gut following the removal of the anti-CSF1R mAb treatment.

### Macrophage depletion indirectly impairs crypt homeostasis

**Fig. 5:**
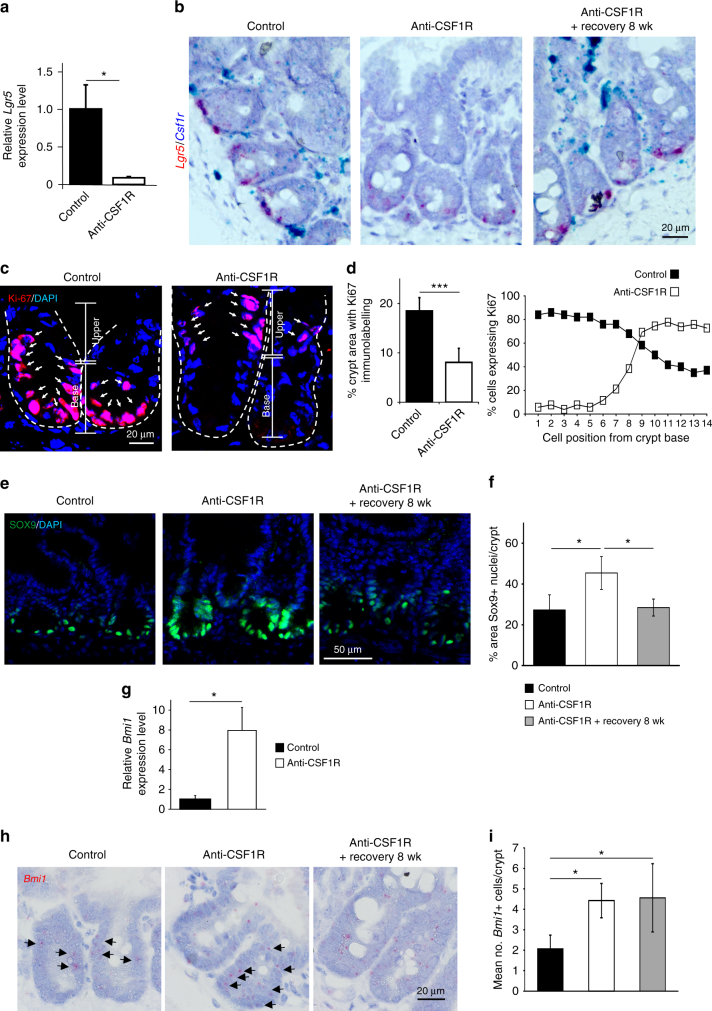
Prolonged anti-CSF1R treatment impairs crypt homeostasis. Mice were treated with anti-CSF1R mAb or control-IgG (control) for 6 weeks before analysis. A parallel group of mice were allowed to recover for 8 wk following anti-CSF1R mAb treatment. **a** Comparison of *Lgr5* mRNA expression in isolated intestinal crypts from anti-CSF1R (open bars) or control-treated (closed bars) mice by RT–qPCR analysis. Bars represent mean ± SEM derived from four mice/group. *P < 0.05, two-tailed unpaired Student’s *t*-test. **b** Two-color mRNA in situ hybridization analysis confirmed a reduction in *Lgr5* mRNA expression (red) at the base of intestinal crypts coincident with the ablation of *Csf1r* mRNA (blue) expressing macrophages. Representative images from three mice/group are shown. Scale bar, 25 µm. **c** Detection of proliferating Ki-67^+^ cells (red) in intestinal crypts by IHC. Sections counterstained with DAPI to detect nuclei (blue). Broken lines indicate the crypt epithelial cell layer boundary. Scale bar, 20 µm. **d** Morphometric analysis of (left) the magnitude of Ki-67-specific immunostaining in intestinal crypts, and (right) the distribution of Ki-67-expression within the crypt. Data represent mean ± SD derived from 5 to 9 sections from four mice/group. ***P < 0.001, two-tailed unpaired Student’s *t-*test. **e** IHC detection of SOX9 expression (green) in intestinal crypts. Sections were counterstained with DAPI to detect cell nuclei (blue). Scale bar, 50 µm. **f** Morphometric analysis confirmed that following prolonged CSF1R blockade the % area of the crypts with SOX9 + cell nuclei was significantly increased. Bars represent mean ± SD derived from four mice/group with 50–100 crypts/mouse, and are representative of data from two independent experiments. *P < 0.05, one-way ANOVA with Tukey’s post hoc test. **g** RT–qPCR analysis of *Bmi1* mRNA expression in isolated intestinal crypts from anti-CSF1R or control-treated mice. Bars represent mean ± SEM derived from four mice/group. *P < 0.05, two-tailed unpaired Student’s *t*-test. **h** mRNA in situ hybridization analysis revealed an increase in the abundance of *Bmi1*-expressing cells (red, arrows) in the intestinal crypts of anti-CSF1R-treated mice. Scale bar, 20 µm. **i** Mean no. *Bmi1*-expressing cells/crypt. Bars represent mean ± SD derived from four mice/group with 40–50 crypts/mouse. *P < 0.05, one-way ANOVA with Tukey’s post hoc test

As noted earlier, Paneth cells help maintain the Lgr5^+^ intestinal stem cells in the crypt niche through the provision of homeostatic factors^2^. Following prolonged CSF1R blockade a reduction in *Lgr5* mRNA expression by the intestinal stem cells at the base of the intestinal crypts was observed (Fig. 5a, b). The effects of prolonged CSF1R blockade on *Lgr5* expression were also transient and returned to similar levels as observed in control mice following the 8 wk recovery period after anti-CSF1R mAb treatment (Fig. 5b). The reduced *Lgr5* expression coincided with a significant reduction in Ki-67^+^ proliferating cells in the base of the intestinal crypt (Fig. 5c, d). Independent studies have shown that a reserve stem cell population within the intestinal crypts can compensate for the complete loss of *Lgr5*-expressing cells^28,29^. In the steady state these cells are mostly restricted to the “+4” cell position within the crypt adjacent to the upper most Paneth cell^30^. These reserve intestinal stem cells express SOX9 and BMI1 and expand during crypt injury to help maintain gut epithelial homeostasis^28,29^. Consistent with this activity, following the reduction in *Lgr5* expression after prolonged CSF1R blockade, cell proliferation (Ki-67^+^ cells) was displaced to the upper crypt region in tissues from anti-CSF1R-treated mice (Fig. 5c, d). A significant increase in the abundance of SOX9^+^ cell nuclei (Fig. 5e, f) and *Bmi1* mRNA expression (Fig. 5g–i) was also observed in the crypts after prolonged CSF1R blockade, suggesting expansion of the reserve intestinal stem cell pool.

The expansion of the reserve intestinal stem cells following CSF1R blockade is consistent with our previous demonstration that villous length was significantly longer in the small intestines of mice treated with anti-CSF1R mAb^13^. The large intestine lacks Paneth cells, but in the colon regenerating islet-derived family member 4 (REG4)-expressing deep secretory cells appear to maintain crypt homeostasis^31^. In the current study, colonic crypt length was also significantly extended in the large intestines of anti-CSF1R mAb mice, implying a similar role for macrophages in the maintenance of the colonic crypt niche (Supplementary Fig. 8).

**Fig. 6:**
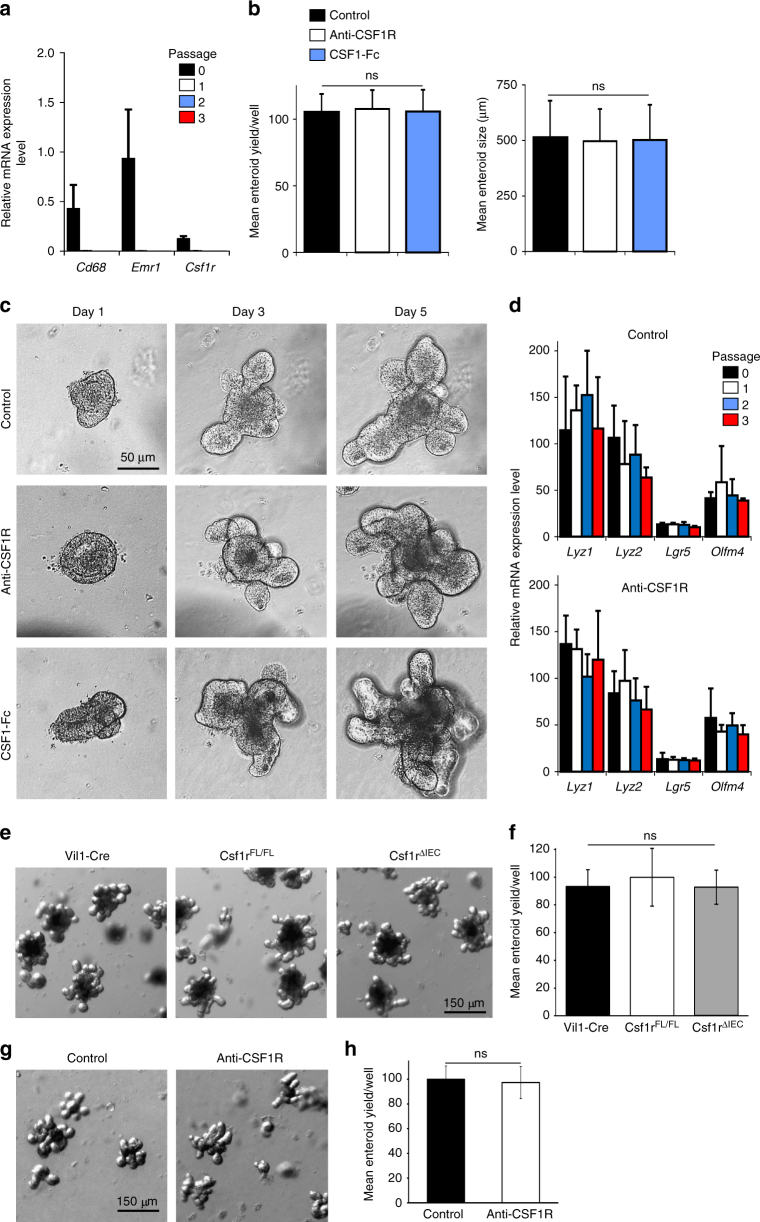
Anti-CSF1R-treatment or CSF1-stimulation do not directly affect crypt homeostasis. **a** RT–qPCR analysis shows enteroids prepared from the small intestines of untreated C57BL/6J mice express negligible levels of *Cd68*, *Emr1*, and *Csf1r*. Expression of these genes is undetectable after passage. Data represent mean ± SEM from triplicate cultures. **b**, **c** Enteroids were prepared from the small intestines of C57BL/6J mice and at passage 3 were cultivated in the presence or absence of anti-CSF1R mAb (10 µg/ml), control rat IgG (10 µg/ml) or CSF1-Fc (500 ng/ml). **b** Mean enteroid yield and size at day 5 after treatment. Data represent mean ± SD from eight wells/treatment. Enteroid yield; NS Kruskal–Wallis one-way ANOVA. Enteroid size; NS one-way ANOVA with Tukey’s post hoc test. **c** Representative morphology of the enteroids at intervals after treatment. Scale bar, 50 µm. **d** RT–qPCR analysis shows the expression of *Lyz1*, *Lyz2*, *Lgr5*, and *Olfm4* in enteroids was unaffected by treatment with anti-CSF1R mAb. Data represent mean ± SEM from triplicate cultures. **e** Representative images of day 7 enteroids prepared from the crypts of Csf1r^ΔIEC^, Vil1-Cre and Csf1r^FL/FL^ mice. Scale bar, 150 µm. **f** The mean yield of enteroids prepared from the crypts of Csf1r^ΔIEC^, Vil1-Cre and Csf1r^FL/FL^ mice on day 7 of cultivation was similar. Data represent mean ± SD from enteroids prepared from 3 mice/group, 9 wells/mouse. ns, one-way ANOVA with Tukey’s post hoc test. **g** Representative images of day 5 enteroids prepared from the crypts of mice treated with anti-CSF1R mAb or control-IgG (control) for 6 weeks. Scale bar, 150 µm. **h** The mean yield of enteroids prepared from the crypts of mice from each group was similar. Data represent mean ± SEM from 14 to 16 wells of organoids from four mice/group. NS two-tailed unpaired Student’s *t*-test

To further eliminate direct roles of CSF1R signaling in the gut epithelium, in vitro enteroids (comprising only of epithelial cell lineages) were prepared from small intestinal crypts of C57BL/6J wild-type mice and treated with either anti-CSF1R mAb, or recombinant CSF1-Fc. Low levels of expression of macrophage-associated genes and *Csf1r* were detected in enteroids freshly prepared from the intestine (passage 0; Fig. 6a), but expression of these genes was undetectable after subsequent passages (passages 1–3; Fig. 6a). Neither anti-CSF1R treatment, nor addition of exogenous CSF1, had any significant effect on the growth of these enteroids throughout multiple serial passages (Fig. 6b, c), nor the expression of lysozyme by Paneth cells, and *Lgr5* and *Olfm4* (encoding olfactomedin-4) by the intestinal stem cells within them (Fig. 6d). These data also suggest that the growth factors added to these cultures to sustain the enteroids in vitro can substitute for the in vivo requirement for macrophages. Consistent with the absence of *Csf1r* mRNA expression by any gut epithelial cell lineage, the specific ablation of *Csf1r* only in the gut epithelium similarly did not affect the growth of enteroids prepared from Csf1r^ΔIEC^ mice, when compared to those prepared from Vil1-Cre or Csf1r^FL/FL^ mice transgenic control mice (Fig. 6e, f).

Enteroids prepared from crypts of mice depleted of Lgr5-expressing intestinal stem cells grow with a similar efficiency as those prepared from control mice due to the increased activity of the reserve intestinal stem cell population^28^. In the current study, the growth of enteroids prepared from the crypts from mice treated with anti-CSF1R mAb was similar to those derived from control-treated mice (Fig. 6g, h). This suggests that the reserve intestinal stem cell population is similarly able to compensate for the reduction in *Lgr5*-expressing stem cells in the crypt following prolonged CSF1R blockade.

The functions of the crypt-associated macrophages could be mediated indirectly, through impacts observed on Paneth cells, or directly by acting upon the Lgr5^+^ stem cells. Several recent papers implicate macrophages as the source of Wnt family members that control epithelial differentiation following injury^32,33^. We therefore compared the expression of mRNA encoding important genes involved in Wnt-signaling and the homeostatic maintenance of the intestinal crypts in microarray datasets derived from distinct mononuclear phagocyte populations isolated from the small intestine. This analysis revealed that small intestinal macrophages specifically expressed *Wnt4* and *Rspo1* (R-spondin, the ligand for Lgr5), and IHC analysis confirmed the expression of Wnt4 by CD68^+^ macrophages in the gut wall (Supplementary Fig. 9).

### Macrophage depletion indirectly impairs M-cell differentiation

**Fig. 7:**
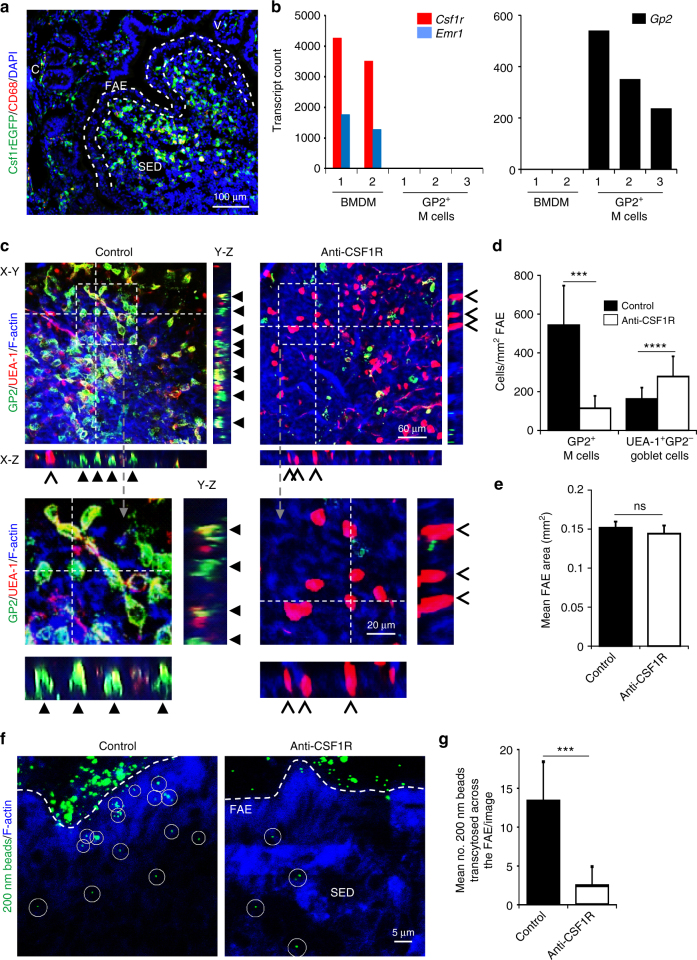
M cells are dramatically reduced in Peyer’s patches after prolonged CSF1R blockade. **a** Histological analysis of *Csf1r*-EGFP mice shows CSF1R (green) is not expressed by epithelial cells in Peyer’s patches. FAE, follicle-associated epithelium; SED, subepithelial dome; dotted lines indicate the FAE boundary. Scale bar, 100 µm. **b** Expression of *Csf1r* (middle panel) and *Gp2* in individual deep CAGE sequence datasets of mouse bone marrow-derived macrophages (BMDM) and M cells^25^. *Csf1r* mRNA is undetectable in M cells. **c**–**g** Mice were treated with anti-CSF1R mAb or control-IgG (control) for 6 weeks before subsequent analysis. **c** Peyer’s patches were whole-mount immunostained to detect GP2 (green), UEA-1 (red) and f-actin (blue). Closed arrows, GP2^+^ M cells with characteristic basolateral pockets; chevrons, GP2-UEA-1^+^ goblet cells. The positions of the X–Z and Y–Z projections of the FAE are indicated by the broken lines in the X–Y images. Scale bar, 60 µm. Boxed areas are shown below in higher magnification. Scale bar, 20 µm. **d** Morphometric analysis showed that the density of GP2^+^ M cells was significantly reduced after prolonged CSF1R blockade, whereas the density of GP2^−^UEA-1^+^ goblet cells was significantly increased. Data are mean ± SD derived from 10 to 13 mice/group, 1 to 9 FAE/mouse from two independent experiments. ***P < 0.001, Mann–Whitney *U*-test; ****P < 0.001, two-tailed unpaired Student’s *t*-test. **e** Morphometric analysis showed that FAE size was unchanged after prolonged CSF1R blockade. Data are mean ± SEM derived from 10 to 13 mice/group, 1 to 9 FAE/mouse from two independent experiments. NS Mann–Whitney *U*-test. **f**, **g** The uptake of particulate antigen (fluorescent beads, green) into the Peyer’s patches was significantly impaired in anti-CSF1R-treated mice. Circles highlight individual beads in the SED. Sections counterstained to detect F-actin (blue). Scale bar, 5 µm. **g** The number of beads transcytosed across the FAE was significantly reduced in anti-CSF1R-treated mice. Data represent mean ± SD from 6 to 12 sections from 4 mice/group. ***P < 0.001, Mann–Whitney *U*-test

The indirect effect of prolonged CSF1R blockade on intestinal crypt homeostasis disturbs the subsequent differentiation of several epithelial cell lineages. For example, the density of goblet cells in the gut epithelium was significantly increased in *Csf1r*^−/−^ mice or *Csf1*^op/op^ mice^10^ or following prolonged CSF1R blockade^13^. M cells are a unique subset of epithelial cells which are specialized for the transcytosis of microorganisms and particulate antigens across the gut epithelium into the Peyer’s patches^18^. M cells do not express *Csf1r* or translate CSF1R protein (Fig. 7a, b, Supplementary Fig. 10), but since they also derive from *Lgr5*-expressing intestinal stem cells^17^ we reasoned that prolonged CSF1R blockade could indirectly affect M-cell differentiation. Mature, glycoprotein 2 (GP2)-expressing M cells with characteristic basolateral pockets were abundant in the FAE of Peyer’s patches from control-treated mice, but their density was reduced after prolonged CSF1R blockade (Fig. 7c, d; *P* < 0.001). The size of the FAE was not affected by prolonged CSF1R blockade (Fig. 7e). Like the other effects of anti-CSF1R-mAb treatment, M cell differentiation was restored within 8 wk following cessation of treatment (Supplementary Fig. 11A, B), coincident with the restoration of the macrophages and crypt homeostasis. M cell density was unaffected in the FAE of Peyer’s patches from Csf1r^ΔIEC^ mice (Supplementary Fig. 11C, D) excluding a direct role for CSF1R signaling in the gut epithelium in M cell differentiation. Consistent with our previous study^13^, a significant increase in the density of goblet cells was observed in the FAE of Peyer’s patches in the small intestine after prolonged CSF1R blockade (Fig. 7c, d; recognized by their morphology, binding of UEA-1 and absence of GP2; *P* < 0.001). A significant increase in goblet cell density was also observed in the colons of the same mice (Supplementary Fig. 8).

We next determined whether the reduced density of GP2^+^ M cells in anti-CSF1R-treated mice correlated with a reduced functional ability to acquire particulate antigens from the gut lumen. The assessment of the uptake of fluorescent latex beads injected into ligated intestinal loops is a useful quantitative method to compare the functional activity of M cells in vivo^20,34,35^. In the Peyer’s patches of control-treated mice numerous beads had been transcytosed across the FAE into the subepithelial dome (SED) region (Fig. 7f). However, following prolonged CSF1R blockade this ability was reduced demonstrating that the functional capacity of M cells to acquire particulate antigens from the gut lumen was adversely affected (Fig. 7f, g; *P* < 0.001).

**Fig. 8:**
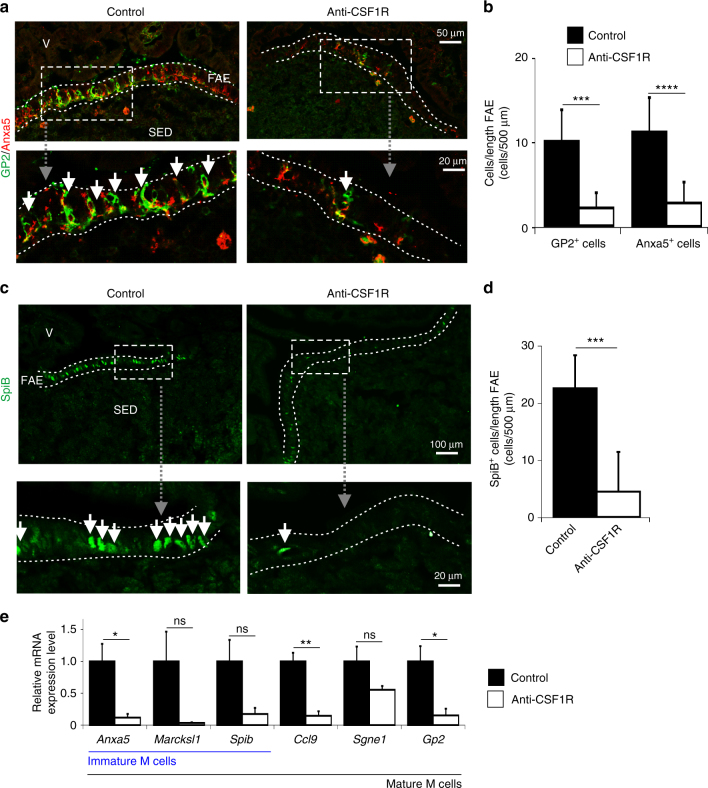
Loss of immature and mature M-cell marker expression in Peyer’s patches after prolonged CSF1R blockade. Mice were treated with anti-CSF1R mAb or control-IgG (control) for 6 weeks before subsequent analysis. **a** Detection of Anxa5 (red) and GP2 (green) in the follicle-associated epithelium (FAE) by IHC. Scale bar, upper panels, 50 µm. Scale bar, lower panel, 20 µm. **b** Morphometric analysis of the number of GP2^+^ and Anxa5^+^ M cells in the FAE. SED, subepithelial dome. Data represent mean ± SD from 1 to 7 FAE from 8 mice/group. ***, *P* < 0.001, two-tailed unpaired Student’s *t*-test; ****P < 0.001, Mann–Whitney *U*-test. **c** IHC analysis of SpiB expression (green) in the FAE. Dotted line in *A* & *C* shows the FAE boundary. Scale bar, upper panels, 100 µm. Scale bar, lower panel, 20 µm. **d** Morphometric analysis of the number of SpiB^+^ cells in the FAE. Data represent mean ± SD from 6 to 9 FAE from four mice/group. ***, *P* < 0.001, Mann–Whitney *U*-test. **e** RT–qPCR analysis shows the expression of immature (*Anxa5*, *Marcksl1*, and *Spib*) and mature (*Ccl9*, *Sgne1*, and *Gp2*) M cell-related genes was reduced in Peyer’s patches after prolonged CSF1R blockade. Bars represent mean ± SEM derived from four mice/group. *P < 0.05; **P < 0.01, two-tailed unpaired Student’s *t*-test. NS not significant

The differentiation of Lgr5^+^ stem cell-derived daughter cells into functionally mature M cells requires additional stimulation from the mesenchymal stromal cells within the SED region beneath the FAE. In the GALT the production of the cytokine, receptor activator of NF-κB ligand (RANKL), by these subepithelial mesenchymal stromal cells is the critical factor which stimulates the differentiation of RANK-expressing (the receptor for RANKL) enterocytes into M cells^35^. This differentiation process can be divided into distinct stages based on the expression particular M-cell markers^18,20^. Immature, differentiating M cells express annexin A5 (Anxa5) and Marcksl1. The intrinsic expression of the ETS transcription factor SpiB by these differentiating M cells is critical for their subsequent differentiation into functionally mature M cells, which in addition to GP2, also express CCL9 and SGNE1^18,20^. Prolonged CSF1R blockade affected M-cell development prior to their differentiation into the immature stage as the expression of all of these immature and mature M cell markers was reduced by the treatment (Fig. 8).

**Fig. 9:**
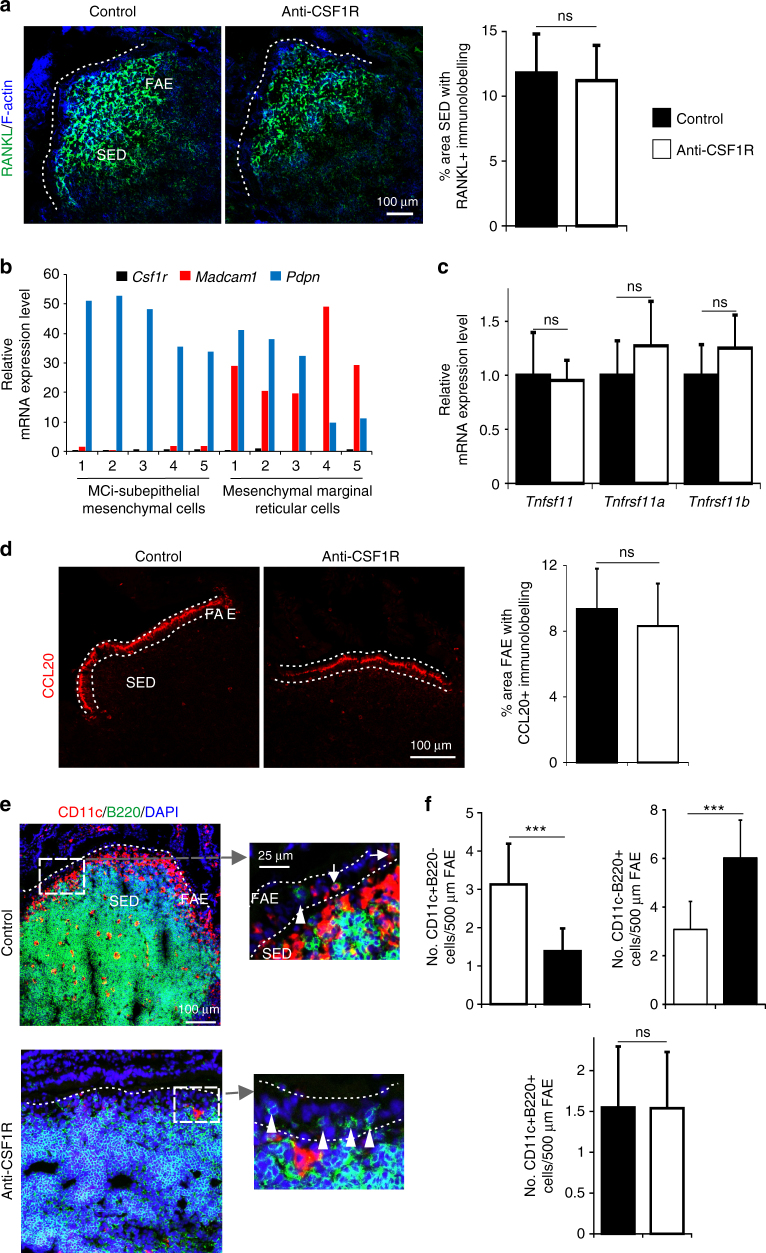
Prolonged CSF1R blockade does not affect RANKL or CCL20 expression in Peyer’s patches. **a** IHC analysis shows no observable difference in the expression or distribution of RANKL on subepithelial dome (SED) stromal cells after anti-CSF1R mAb treatment. Scale bar 100 µm. Morphometric analysis (histogram, right) confirmed the magnitude of the RANKL-specific immunostaining in the SED of Peyer’s patches from each group of mice was similar. Data represent mean ± SD from 3 to 4 mice/group and 1 to 3 SED regions/mouse. NS Mann–Whitney *U*-test. **b** Expression of *Csf1r* (black), Madcam1 (red) and *Pdpn* (blue) in individual mRNA sequencing datasets of mouse “M cell-inducing-” (MCi)-subepithelial mesenchymal cells and mesenchymal marginal reticular cells^37^. **c** RT–qPCR analysis shows no significant difference in the expression of *Tnfsf11* (RANKL), *Tnfrsf11a* (RANK), or *Tnfrsf11b* (OPG) mRNA in Peyer’s patches following anti-CSF1R treatment. Bars represent mean ± SEM derived from four mice/group. NS not significant, two-tailed unpaired Student’s *t*-test. **d** IHC analysis shows no observable difference in the expression of CCL20 in the follicle-associated epithelium (FAE) after anti-CSF1R mAb treatment. Scale bar, 100 µm. Morphometric analysis (histogram, right) confirmed the magnitude of the CCL20-specific immunostaining in Peyer’s patches from each group of mice was similar. Data represent mean ± SD from 3 to 9 FAE from four mice/group. NS two-tailed unpaired Student’s *t*-test. **e** IHC analysis of the distribution of CD11c^+^ (red) and B220^+^ (green) cells in the Peyer’s patches after anti-CSF1R mAb treatment (scale bar, 100 µm). Boxed areas are shown adjacently in higher magnification (scale bar, 25 µm). Arrows indicate CD11c^+^B220^+^ “M cell-inducing” B cells. Arrow-heads indicate CD11c^−^B220^+^ B cells. Sections counterstained with DAPI to detect nuclei (blue). **f** After anti-CSF1R treatment morphometric analysis showed that the number of CD11c^+^B220^−^ mononuclear phagocytes in the FAE was significantly decreased (left-hand panel), the number of CD11c^−^B220^+^ B cells was increased (right-hand panel), but the number of CD11c^+^B220^+^ “M cell-inducing” B cells was unchanged (lower panel). Dotted line indicates the FAE boundary. Data represent mean ± SD from 1–11 FAE from four mice/group. ***P < 0.001, two-tailed unpaired Student’s *t*-test. NS not significant

Although CSF1 can regulate RANKL expression by osteoclasts^36^, the effects of prolonged CSF1R blockade on M-cell status observed here were not due to impaired RANKL-RANK stimulation. High levels of RANKL were detected on the subepithelial mesenchymal stromal cells in the Peyer’s patches of anti-CSF1R-treated and control-treated mice (Fig. 9a). Consistent with this observation, independent mRNA sequencing data from subepithelial mesenchymal stromal (podplanin^+^MAdCAM-1^−^) cells and mesenchymal marginal reticular (podplanin^+^MAdCAM-1^+^) cells enriched from Peyer’s patches^37^ indicates that neither cell population expresses *Csf1r* (Fig. 9b). The expression of *Tnfsf11* (encoding RANKL), *Tnfrsf11a* (encoding RANK), and *Tnfrsf11b* (encoding the RANKL decoy receptor osteoprotegerin, OPG) in Peyer’s patches was also unaffected by prolonged CSF1R blockade (Fig. 9c). The CCL20-mediatiated attraction of CD11c-expressing B cells towards the FAE has also been shown to aid the maturation of M cells from immature Anxa5^+^ precursors^34,38^. However, CCL20 expression in the FAE (Fig. 9d) and the attraction of “M cell-inducing” CD11c^+^ B cells towards it (Fig. 9e, f) were likewise unaffected by prolonged CSF1R blockade. These data are consistent with the demonstration that M cell development was adversely affected prior to their differentiation into the immature Anxa5^+^ stage (Fig. 8).

Taken together, these data demonstrate that the effects of prolonged CSF1R blockade on M-cell development were due to disturbances in crypt homeostasis. These data reveal a previously unrecognized, indirect requirement for macrophages in the maintenance of M cells in Peyer’s patches through their essential role in the constitutive maintenance of Paneth cells and Lgr5^+^ stem cells in the FAE-associated crypts.

## Discussion

Here we show that crypt-associated macrophages are constitutively required to maintain the crypts in the small intestine. Their depletion following prolonged CSF1R blockade affects the differentiation of Paneth cells and Lgr5^+^ stem cells in the crypt, notably resulting in a change in the balance between goblet cell and M cell differentiation. These data suggest that modification of the phenotype or abundance of macrophages in the gut wall, for example after pathogen infection, could adversely affect the development of the intestinal epithelium and the ability to sample particulate antigen from the gut lumen.

In the small intestine the Lgr5^+^ stem cells within the base of the crypts are interspersed between terminally differentiated Paneth cells. The Paneth cells provide essential stimuli for the constitutive maintenance of the Lgr5^+^ stem cells including Wnt3A, EGF, TGFα and the Notch ligand Dll4^2,39,40^, and the loss of Paneth cells results in a significant reduction in Lgr5^+^ stem cells^2^. In the current study, at least part of the effect of macrophage depletion probably occurs through the impact on Paneth cells, which is likely responsible for the reduction of Lgr5^+^ stem cells. However, it is possible that the macrophages also interact directly with the stem cells, as the proliferation of Lgr5^+^ stem cells is not entirely Paneth cell dependent. In Paneth cell deficient *Atoh1*^−/−^ mice some Lgr5^+^ stem cells can survive and proliferate^41^. Furthermore, the specific deletion of Wnt signals only in the gut epithelium or in subepithelial myofibroblasts does not affect the status of Paneth cells or Lgr5^+^ stem cells, indicating the presence of an alternative cellular source of support^42,43^. Wnt-signaling in the intestinal stem cell niche requires direct cell-to-cell contact^39^. Given the tight association of macrophages with the intestinal crypt, we hypothesize that in the specific absence of Paneth cells, factors derived from the crypt-associated macrophages are able to provide homeostatic support to the Lgr5^+^ stem cells.

In contrast to conclusions from previous reports^9,10^, we show that prolonged CSF1R blockade did not lead to the loss of Paneth cells, as cells with abundant secretory cytoplasmic granules were evident in the crypts of treated mice. Instead, we show that crypt-associated macrophages provide factors which help to maintain Paneth cell differentiation. Paneth cells release antimicrobial products such as lysozyme and α-defensins alongside homeostatic factors such as Wnt3 which are essential for the maintenance of the Lgr5^+^ intestinal stem cells^2^. In the absence of the crypt-associated macrophages the expression of lysozyme and Wnt3 by Paneth cells was significantly reduced. The release of antimicrobial products by Paneth cells helps to prevent bacterial translocation across the gut epithelium and protects the crypts from bacterial infection. Whereas Paneth cell-derived α-defensins have selective antimicrobial activity against pathogenic bacteria, they have limited or no activity against commensal bacteria^44^. Defects in Paneth cells or their production of antimicrobial factors can result in intestinal microbiota dysbiosis, expansion of pathogenic noncommensals and susceptibility to enteric infections^44,45^. The expression of *Defa1* by Paneth cells was unaffected by prolonged CSF1R blockade, implying that the protection of the crypts against pathogenic bacteria was maintained. However, the reduced Paneth cell-derived lysozyme expression may cause microbiota dysbiosis and the expansion of certain commensal populations such as *Escherichia* *coli*^46^.

The factors produced by the crypt-associated macrophages which provide this essential support to Paneth cells are not known. The clear apposition of macrophages with the intestinal crypt stem cells strongly suggests a trophic interaction, perhaps akin to their relationship with haemopoietic stem cells in the bone marrow^47,48^, or with osteoblasts on the surface of bone^49^. Wnt signals can separately stimulate both Lgr5^+^ stem cells and Paneth cells^40^, and Wnt-signal expression by macrophages is widely documented^32,50^. Bone morphogenetic protein signaling is also essential for the maintenance of the Lgr5^+^ stem cell population^51^, and there may be parallels with the trophic interactions between macrophages and neurons in the *muscularis externa*^15,16^. Indeed, the same reciprocal relationship between macrophages and CSF1-producing cells may occur, since Paneth cells have been reported to be sites of CSF1-synthesis^52^. Paneth cells may also provide EGF to the Lgr5^+^ stem cells^2^, and here also, CSF1-dependent macrophages could provide an additional source of growth factor^53^. In overview, it is unlikely that the trophic function of crypt-associated macrophages relies on a single factor. In support of this we found that the expression of two candidate factors, *Wnt4* and *Rspo1* was enriched in small intestinal macrophages implying a potential role. Since R-spondin 1 is a Wnt agonist, the ligand for Lgr5^54^ and is an essential factor required for the in vitro cultivation of enteroids^1^ it is plausible that the macrophages interact directly with, and provide homeostatic support to, both Paneth cells and intestinal stem cells.

Our data contradict the view that Paneth cells express functional CSF1R^9,10^. One group has suggested that CSF1R can also be expressed by renal epithelial cells during regeneration in response to injury^55^, but others have demonstrated that in this system the impacts of CSF1 treatment are also mediated through macrophages^56^. The growth of enteroids from *Csf1r*^−/−^ mice has been reported to be impaired^9^. However, in the current study enteroids prepared from Csf1r^ΔIEC^ mice grew equally well, and neither CSF1, nor anti-CSF1R mAb treatment, has any effect in vitro. The reasons for the discrepancies between these studies is uncertain. The published studies relied on the use of a polyclonal anti-CSF1R antibody to detect the protein^10^ and a tamoxifen-inducible Cre to initiate conditional deletion of *Csf1r*^9^. The former might not be specific, while in the latter case, direct effects of tamoxifen on macrophages may not be excluded. Although crypt homeostasis was disturbed following prolonged CSF1R blockade, the growth of enteroids prepared from the intestines of these mice in the current study was equivalent to those from controls. Independent studies have shown that enteroids prepared from the crypts of mice which were depleted of *Lgr5*^+^ intestinal stem cells are similarly able to grow and proliferate due to the expansion of the reserve intestinal stem cell population^28^, which is insensitive to Wnt pertubations^29^. We observed a similar expansion of the SOX9^+^BMI1^+^ reserve intestinal stem cell population following prolonged CSF1R blockade, suggesting that these cells were similarly attempting to restore crypt homeostasis in the absence of macrophages.

Disturbances to the homeostasis of the crypt can significantly affect the proportions of epithelial cell lineages in the gut epithelium. The Lgr5^+^ stem cells produce highly proliferative transit-amplifying daughter cells which differentiate into one of several specialized intestinal epithelial cell lineages depending on the additional stimulation they receive^20,35,57^. These include secretory cell lineages such as enteroendocrine cells, goblet cells, Paneth cells or tuft cells. For example, the indirect effects of prolonged CSF1R blockade on the crypt enhances goblet cell differentiation in the small intestine^10,13^ and colon. The antigen-sampling M cells also derive from the Lgr5^+^ stem cells in the crypts surrounding the FAE which differentiate into mature, functional GP2^+^ M cells upon RANKL-stimulation from subepithelial mesenchymal stromal cells^17,20,35,37^. The density of M cells in Peyer’s patches was reduced after prolonged CSF1R blockade, as was their ability to acquire particulate antigens from the gut lumen. The effects of anti-CSF1R-treatment on the development of M cells were indirect since these cells also do not express CSF1R. Instead we reveal a previously unrecognized, indirect requirement for macrophages in the maintenance of M cells in Peyer’s patches through their essential role in the constitutive maintenance of the FAE-associated crypts.

The link between lamina propria macrophages and M cells provides a potential feedback loop to ensure some coordination of antigen sampling, innate immune defence and antigen presentation. The pharmacological blockade of CSF1R signaling has also been proposed as a means to modulate certain cancers, and inflammatory, autoimmune and bone diseases^58^. Prolonged CSF1R blockade could indirectly compromise intestinal crypt homeostasis and the ability to sample particulate antigens/pathogens from the gut lumen. Paneth cell-dysfunction is evident in patients who have Crohn’s disease (CD) with small intestinal involvement and may be a consequence of inadequate Wnt-ligand stimulation by monocytes^59^. Monocytes from patients with ileal CD display reduced expression of canonical Wnt ligands including Wnt1, Wnt3, and Wnt3a, and Paneth cell function in biopsies from CD patients can be restored in vitro by PBMC from healthy volunteers^59^. Similarly, macrophages have been shown to promote mucosal repair through activation of Wnt-signaling in a mouse model of inflammatory bowel disease^32^. The corollary of the observed impact of anti-CSF1R treatment on intestinal epithelial cell differentiation is that CSF1 treatment has the potential to restore epithelial function following injury, inflammation or chemotherapy, as has already been demonstrated in other organ systems^55,56,60,61^. Efforts to test this hypothesis are ongoing.

## Methods

### Mice

The following mouse strains were used where indicated. Male (6–8 weeks old) *Csf1r*-EGFP reporter “MacGreen” mice^12^ (bred and maintained in-house) were used for all the experiments. Male and female (6–8 weeks old) Vil1-Cre (Tg(Vil1-cre)997Gum/J strain; The Jackson Laboratory, Bar Harbor, ME, USA) and Csf1r^FL/FL^ (B6.Cg-*Csf1r*^tm1.1jwp^/J; The Jackson Laboratory) were used for all the experiments. Male (6–8 weeks old) C57BL/6 J mice (Charles River Laboratories, Tranent, UK) were used for all the experiments. All studies and regulatory licences were approved by the University of Edinburgh’s ethics committee and performed under the authority of a UK Home Office Project Licence. All animals were coded before use in the studies described below.

### In vivo CSF1R blockade

Mice were injected i.p. with 200 µg rat anti-mouse CSF1R monoclonal antibody (mAb; M279; Amgen, Thousand Oaks, CA, USA) three times/week for 6 weeks^6,13^. Rat IgG (I4131; Sigma, Poole, UK) was used as a control. In some experiments a parallel set of mice were treated with anti-CSF1R mAb for 6 weeks, and then allowed to recover for an additional 8 weeks before analysis.

### Immunohistochemistry (IHC)

Details of the sources, clone numbers and concentrations of all the antibodies used for IHC are provided in Supplementary Table 1. For whole-mount staining, Peyer’s patches were fixed with BD Cytofix/Cytoperm (BD Biosciences, Oxford, UK) and immunostained with rat anti-mouse GP2 mAb (MBL International, Woburn, MA). Tissues were subsequently stained with Alexa Fluor 488-conjugated anti-rat IgG Ab (Invitrogen, Paisley, UK), rhodamine-conjugated *Ulex europaeus* agglutinin I (UEA-1; Vector Laboratories Inc., Burlingame, CA) and Alexa Fluor 647-conjugated phalloidin (ThermoFisher Scientific, Paisley, UK).

For analysis of tissue sections, intestinal pieces were snap-frozen in liquid nitrogen or fixed in 4% paraformaldehyde before embedding in optimal cryostat temperature medium (VWR, Leighton Buzzard, UK). Tissue sections (5 µm) were immunostained with the primary antibodies listed in Supplementary Table 1. For the detection of lysozyme in intestinal crypts paraformaldehyde-fixed sections were permeabilized with 50% ethanol for 20 min. before immunostaining with rabbit anti-lysozyme mAb (Abcam, Cambridge, UK). For the detection of SpiB in paraformaldehyde-fixed sections, antigen retrieval was performed with citrate buffer (pH 7.0, 121 °C, 5 min) prior to immunostaining with sheep anti-mouse SpiB polyclonal antibody (R&D Systems, Abingdon, UK). Unless indicated otherwise, following the addition of primary antibody, species-specific secondary antibodies coupled to Alexa Fluor 488 and Alexa Fluor 555 dyes were used (Invitrogen). Sections were counterstained with either Alexa Fluor 647-conjugated phalloidin or DAPI (ThermoFisher), mounted in fluorescent mounting medium (Dako, Ely, UK) and examined using a Zeiss LSM710 confocal microscope (Zeiss, Welwyn Garden City, UK). For quantification of goblet cells in the colon sections were treated with PAS (0.5% pararosaniline, 1% sodium metabisulfite).

### In vivo assessment of M cell-mediated transcytosis

The uptake of 200 nm diameter fluoresbrite YG carboxylate microspheres (Polysciences, Eppelheim, Germany) from the gut lumen was assessed as described^34^. Mice (*n* = 4/group) were anesthetized and ~2 cm segments of small intestine containing a single Peyer’s patch were ligated using nylon filament. The loops were then injected with a 200 µl suspension of 200 nm fluorescent nanoparticles diluted in PBS (~1 × 10^11^ beads/ml). The isolated gut loops were then placed back in the peritoneal cavity. Approximately 1 h later the injected gut loops were excised, washed in 0.5% Tween 20/PBS, fixed in 4% paraformaldehyde/PBS, and embedded in OCT. Frozen sections (5 µm) were counterstained with Alexa Fluor 647-conjugated phalloidin and examined using a Nikon Eclipse E400 fluorescence microscope. Data were collected from 10 sections from two Peyer’s patches/mouse.

### Image analysis

For morphometric analysis, digital microscopy images were analyzed using ImageJ software (http://rsb.info.nih.gov/ij/) as described^62^. Briefly, all images were coded and assessed blindly. Background intensity thresholds were first applied using an ImageJ macro which measures pixel intensity across all immunostained and non-stained areas of the images. The obtained pixel intensity threshold value was then applied in all subsequent analyses. Next, the number of pixels of each color (black, red, green, yellow etc.) were automatically counted and presented as a proportion of the total number of pixels in each area under analysis. In each instance, tissues from 4 to 8 mice/group group were analyzed. In order to analyze the expression of several parameters in each mouse, multiple sections from at least three Peyer’s patches were analyzed. In addition at from each mouse between 30 and 120 individual small intestinal and colonic crypts were analyzed.

### Crypt isolation and in vitro enteroid cultivation

Intestinal crypts were dissociated from mouse small intestine using Gentle Cell Dissociation Reagent (Stemcell Tech, Cambridge, UK). Crypts were subsequently used in mRNA expression analyses or for establishing enteroids by cultivation in Matrigel (BD Bioscience, Oxford, UK) and Intesticult medium (Stemcell Technologies, Cambridge, UK)^63,64^. Briefly, crypts were re-suspended in Intesticult medium at 2 × 10^3^ crypts/ml and mixed 1:1 with Matrigel. Next, 50 µl Matrigel plugs were plated in pre-warmed 24-well plates and allowed to settle, before addition of 600 µl of pre-warmed Intesticult medium and subsequently cultured at 37 °C in a 5% CO_2_ atmosphere. Fresh medium was replaced every 2 d of cultivation. Where indicated, some wells were treated with either 10 µg/ml anti-CSF1R mAb (or 10 µg/ml rat IgG as a control) or 500 ng/ml CSF1-Fc^8^. For each experimental condition, enteroids were cultivated in triplicate and repeated on three independent occasions. After 5 d of cultivation the enteroids were either harvested for mRNA expression analysis as described^64^, or passaged as described^63^.

To passage the enteroids, ice cold DMEM F/12 medium (Life Technologies, Paisley, UK) was added to the wells and the enteroids vigorously pipetted to disrupt the Matrigel plug. The enteroids were then allowed to settle by gravity, the supernatant discarded and then washed 3 more times in ice cold DMEM F/12. Crypts were physically disassociated from the main enteroids bodies by vigorous pipetting through a flame-polished glass Pasteur pipette with the tip stretched to reduce the aperture. The dissociated crypts were then allowed to settle by gravity, counted using a haemocytometer, re-suspended in Intesticult medium and enteroid cultures established as above.

For mRNA extraction, the Matrigel plug containing the enteroids was incubated in 500 µl of Corning Cell Recovery Solution (Corning, Wycombe, UK) for 1 h at 4 °C. Once all the Matrigel was depolymerized, the cell pellet was then homogenized in the RLT buffer provided by the DNeasy Blood and Tissue Kit (Qiagen, Manchester, UK). Total RNA was then extracted using the DNeasy Blood and Tissue Kit according to the manufacturer’s instructions.

### Real-time quantitative PCR (RT–qPCR) analysis of mRNA expression

Total RNA was isolated from tissues and crypts using RNA-Bee (AMS Biotechnology, Oxfordshire, UK) followed by treatment with DNase I (Ambion, Warrington, UK). First strand cDNA synthesis was performed using 1 µg of total RNA and the First Strand cDNA Synthesis kit (GE Healthcare, Bucks, UK) as described by the manufacturer. PCR was performed using the Platinum-SYBR Green qPCR SuperMix-UDG kit (Invitrogen) and the Stratagene Mx3000P real-time qPCR system (Stratagene, CA, USA). The qPCR primers (Supplementary Table 2) were designed using Primer3 software^65^. The cycle threshold values were determined using MxPro software (Stratagene) and normalized relative to *Gapdh*.

### RNA in situ hybridizations

Paraformaldehyde-fixed intestine sections (5 µm in thickness) were processed for RNA in situ hybridization detection using RNAscope^®^ 2.5 HD single (red) or duplex (chromogenic) detection kits according to the manufacturer’s instructions (Advanced Cell Diagnostics, Hayward, CA, USA). Target retrieval and protease digestions were performed for 15 and 30 mins, respectively. The RNAscope^®^ probes used are shown in Supplementary Table 3. Positive and negative control probes were used for each experiment according to the manufacturer’s instructions. For analysis of *Bmi1* expression the duration of amplification step 5 was increased to 1 h.

### Statistical analyses

Details of all group/sample sizes and experimental repeats are provided in the figure legends. Unless indicated otherwise, all data are presented as mean ± SD and differences between groups analyzed by a two-sided Student’s *t*-test. In instances where there was evidence of non-normality (identified by the Kolmogorov–Smirnov test), data were analyzed by a Mann–Whitney *U*-test. Values of *P* < 0.05 were accepted as significant.

### Data availability

The authors declare that all data supporting the findings in this study are available within the article and its Supplementary Information files or from the corresponding author on reasonable request. The following published mRNA expression datasets were also analyzed which are publicly available in the Gene Expression Omnibus (https://www.ncbi.nlm.nih.gov/gds) via the following accession codes: GSE53297; GSE93320; GSE100393. Deep CAGE sequence data from the FANTOM consortium^25^ are available via the following URL: http://fantom.gsc.riken.jp/.

## Electronic supplementary material

Supplementary information(PDF 3233 kb)

Description of Additional Supplementary Files(PDF 82 kb)

Supplementary movie 1
