# Supplementary material for: The role of CSF1R-dependent macrophages in control of the intestinal stem-cell niche
Source: Nat Commun. 2018 Mar 28;9:1272. doi: 10.1038/s41467-018-03638-6 (PMC5871851; doi:10.1038/s41467-018-03638-6)
Supplement: Supplementary file 1 — Supplementary information(PDF 3233 kb) [file 41467_2018_3638_MOESM1_ESM.pdf]

The role of CSF1R-dependent macrophages in control of the intestinal stem-cell niche

Anuj Sehgal, David S. Donaldson, Clare Pridans, Kristin A. Sauter, David A. Hume & Neil A. Mabbott

Supplementary Figures and Tables

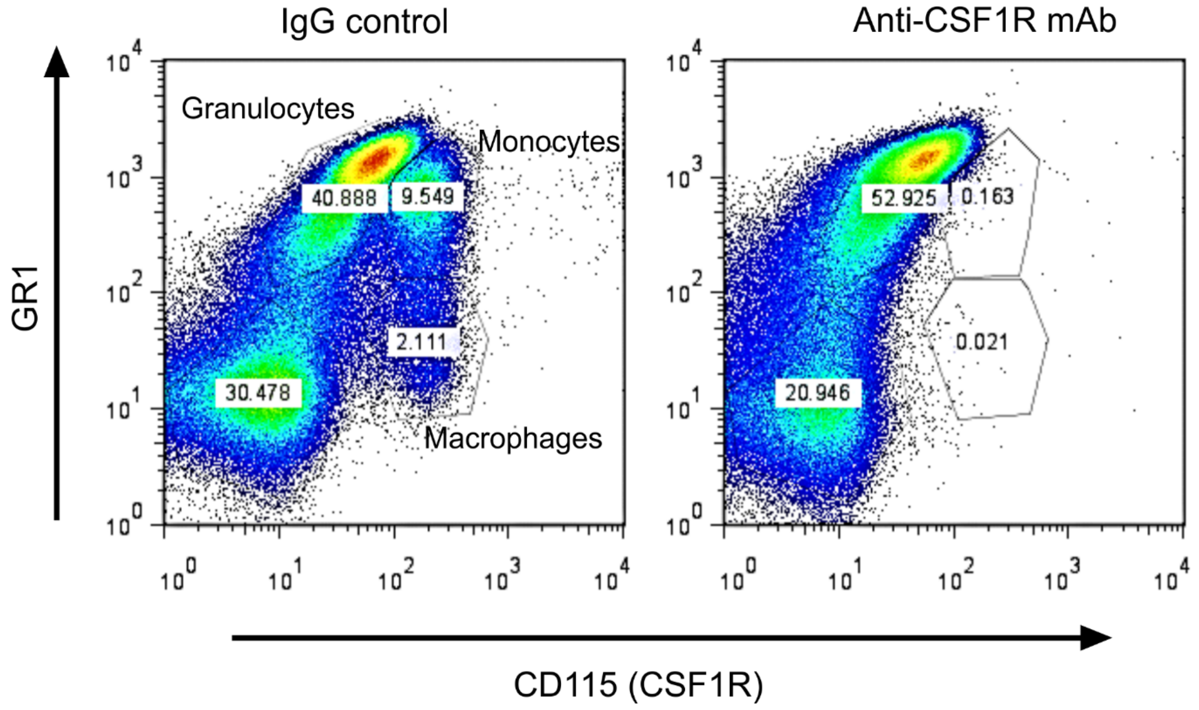

Supplementary Figure 1. Depletion of macrophages in the bone marrow of anti-CSF1R-treated mice. Representative FACS profiles of bone marrow preparations from control rat IgG or anti-CSF1R-treated mice (*n*=3 and *n*=4 mice/group). GR1/CSF1R profiles highlighting the almost complete reduction of cells with monocyte and macrophage characteristics.

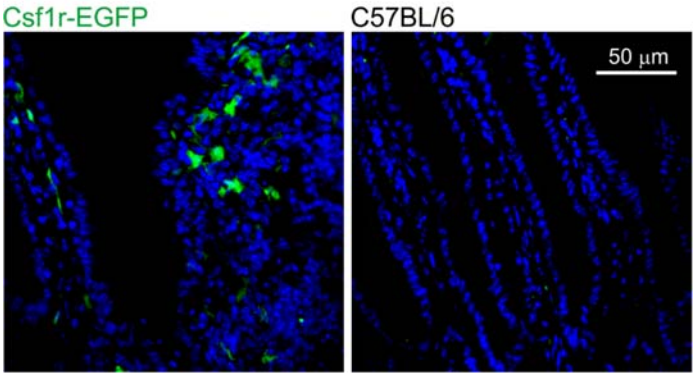

Supplementary Figure 2. Fluorescence microscopical analysis of the intestines from *Csf1r*-EGFP mice and C57BL/6J control mice. Sections were counterstained with DAPI to detect cell nuclei (blue). Scale bar, 50 µm.

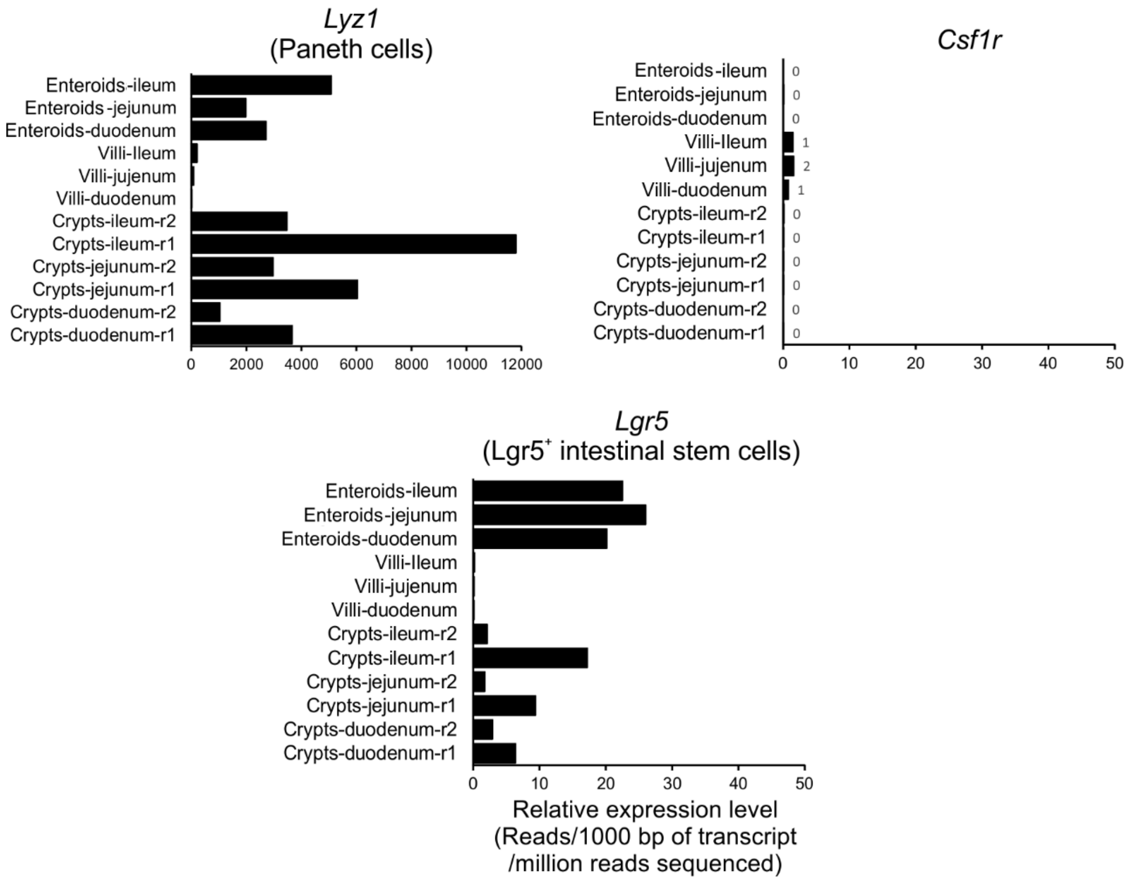

Supplementary Figure 3. Absence of *Csf1r* mRNA expression in isolated small intestinal crypts. Comparison of *Csf1r* mRNA expression in individual data sets from independent mRNA sequencing studies of enteroids, villi and isolated intestinal crypts[1] (GEO data set: GSE53297).

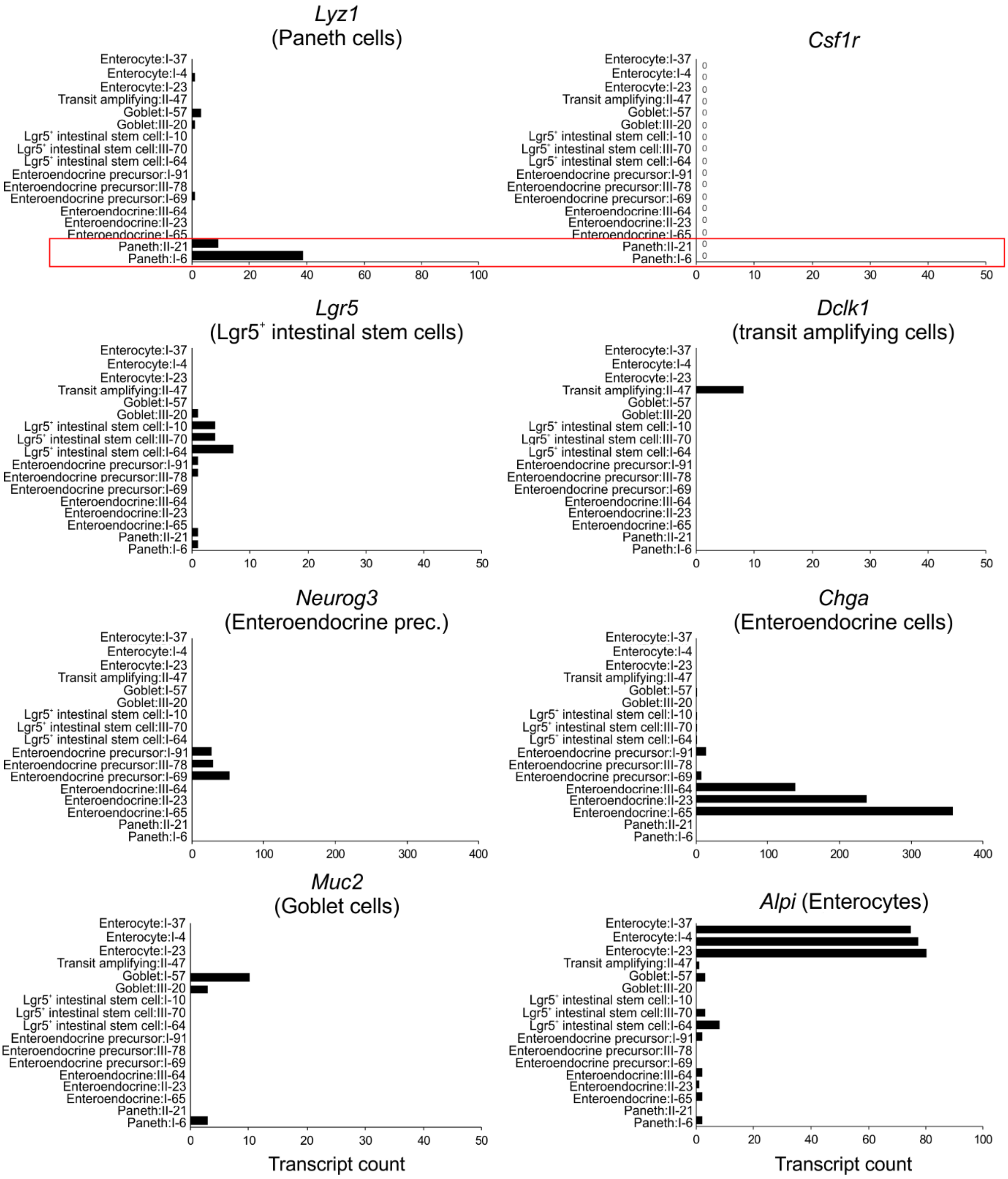

Supplementary Figure 4. Paneth cells and other crypt epithelial cell lineages do not express *Csf1r* mRNA. Comparison of *Csf1r* mRNA expression and other crypt epithelial cell lineage-related genes in data from independent mRNA sequencing studies of individual cell populations derived from intestinal crypts[2].

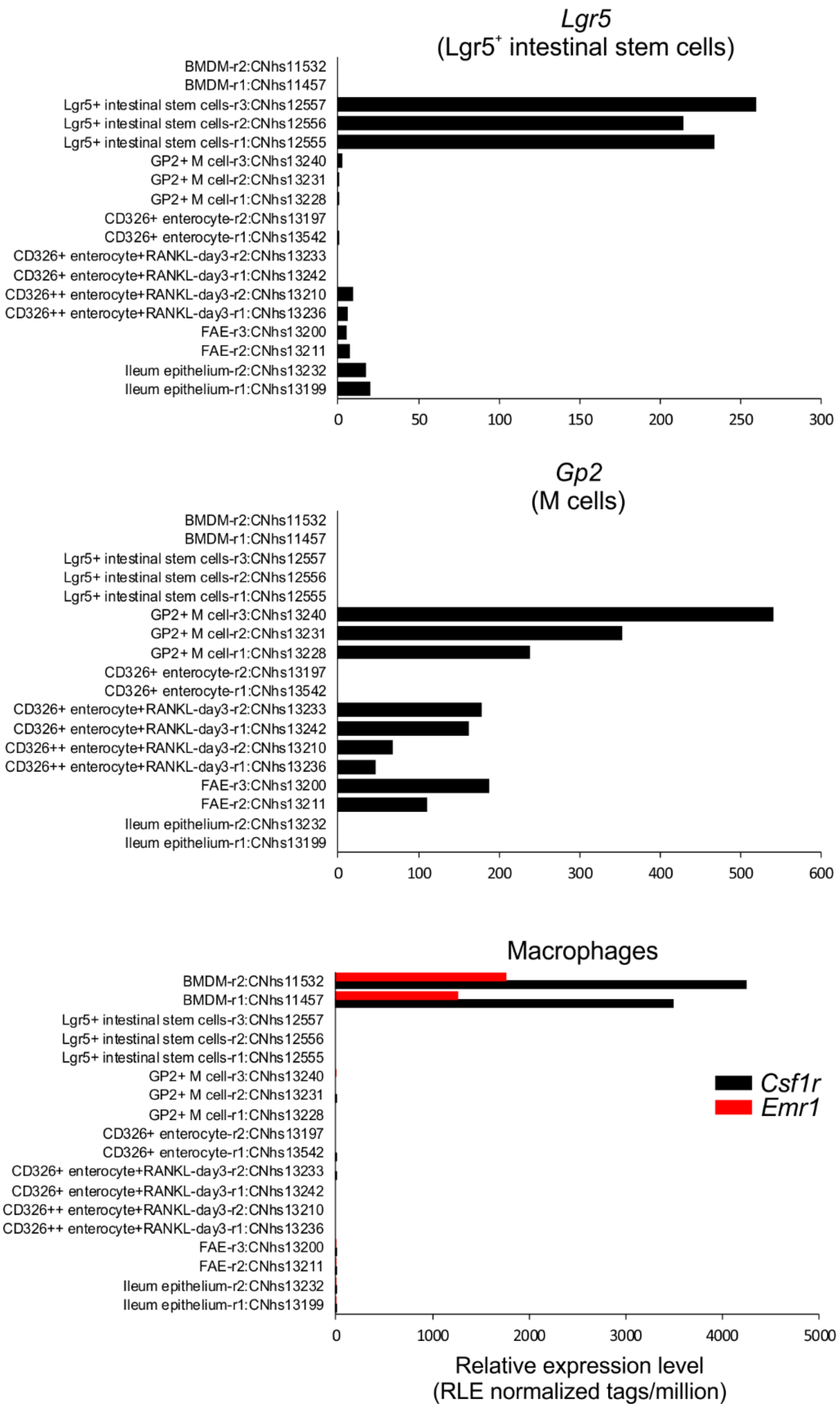

Supplementary Figure 5. Epithelial cell lineages, including M cells, do not express *Csf1r* mRNA. Comparison of *Csf1r*, *Emr1*, *Gp2* and *Lgr5* mRNA expression in individual cell populations in deep CAGE sequence data from the FANTOM5 project of the FANTOM consortium[3] (http://fantom.gsc.riken.jp/zenbu).

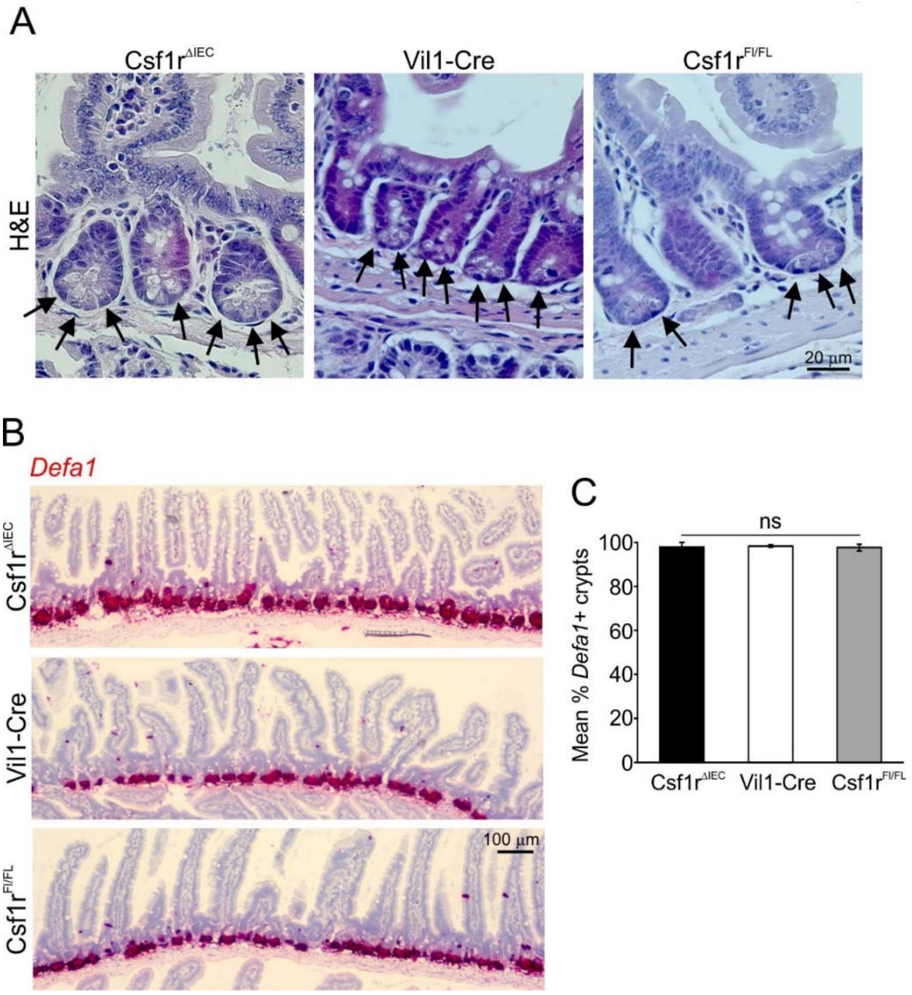

Supplementary Figure 6. Analysis of Paneth cell status in the intestines of Csf1r$^{\Delta IEC}$, Vil1-Cre and Csf1r$^{FL/FL}$ mice. *A*) Analysis of H&E stained intestines revealed that Paneth cells containing secretory granules (arrows) were present in the intestines of mice from each group. Representative images from the intestines of 3 mice/group are shown. Scale bar, 20 µm. (*B*) RNA *in situ* hybridisation analyses showed that *Defa1* mRNA (red) was abundantly expressed in the intestinal crypts of Csf1r$^{\Delta IEC}$, Vil1-Cre and Csf1r$^{FL/FL}$ mice. Representative images from the intestines of 3 mice/group are shown. Scale bar, 100 µm. (*C*) Morphometric analysis confirmed that the % crypts with *Defa1*-expressing Paneth cells was similar in the crypts of mice from each group. Data were obtained from 3 mice/group and 98-100 crypts/mouse. ns, one-way ANOVA with Tukey's post-hoc test.

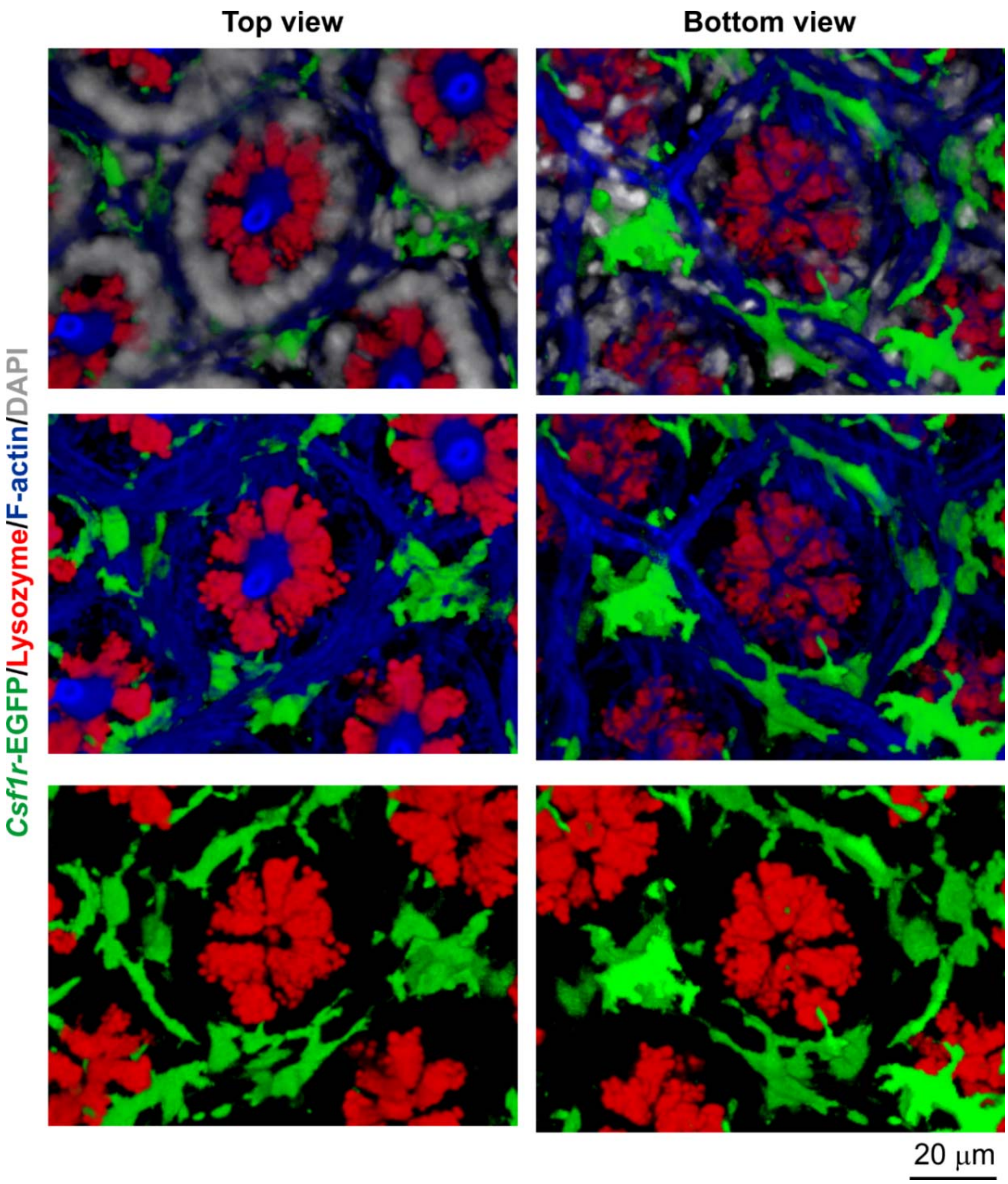

Supplementary Figure 7. Paneth cells do not express CSF1R. Representative whole-mount IHC analysis of intestinal crypts in the intestines of untreated *Csf1r*-EGFP mice shows the lysozyme expressing Paneth cells (red) do not express CSF1R (EGFP, green). In the crypt EGFP-expression was only detected in cells with macrophage morphology. Tissues are counterstained to detect F-actin (blue) and cell nuclei (DAPI, white/grey). Left-hand panels show crypts viewed from the lumenal (upper) surface. Right-hand panels show the same crypts viewed from the serosal (bottom) surface. Scale bar, 20 µm.

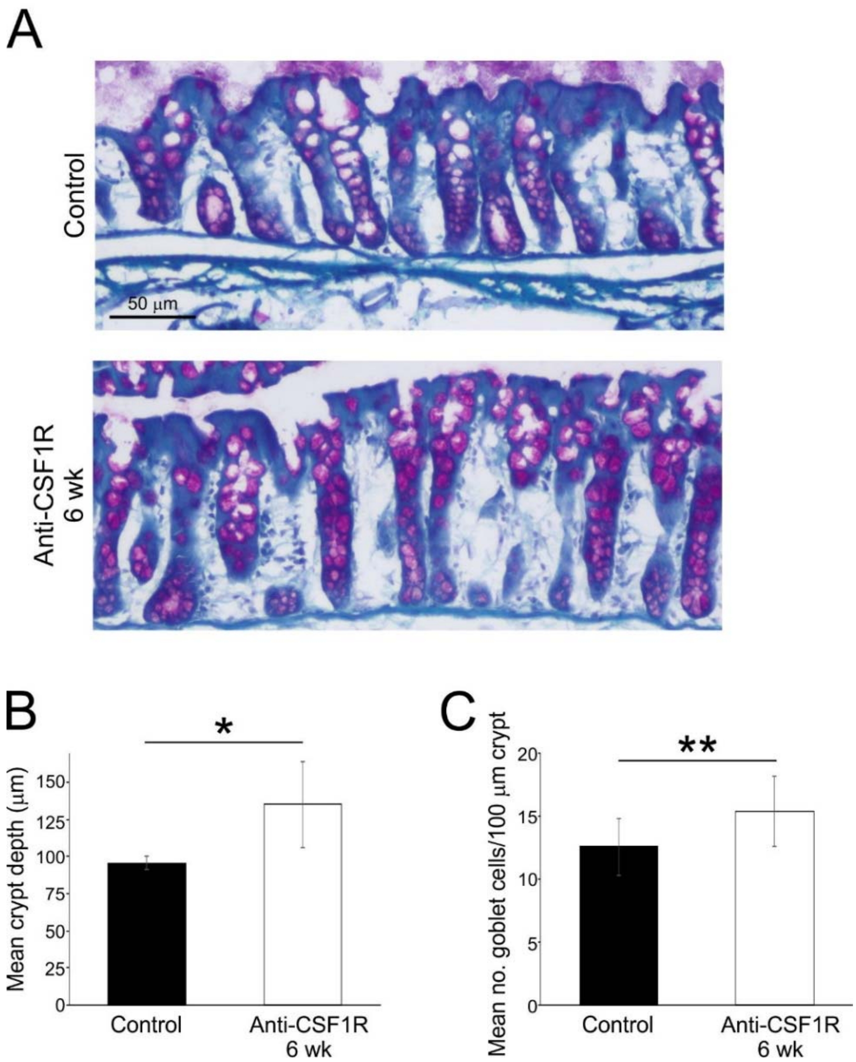

Supplementary Figure 8. Effect of prolonged CSF1R-blockade in the colon. Mice were treated with anti-CSF1R mAb or control-IgG (control) for 6 weeks before colons from 4 mice from each group were removed for analysis. (A) Histological analysis of PAS-stained sections from the colons of mice from each treatment group. Representative images from 4 mice/group, from 3 independent experiments are shown. Scale bar, 50 µm. (B) Morphometric analysis revealed that crypt depth was significantly longer following prolonged CSF1R-blockade. Data were obtained from 4 mice/group and 36-73 crypts/mouse. (C) A significant increase in goblet cell density was also observed in the colonic crypts following prolonged CSF1R-blockade. Data are derived from 4 mice/group and 29-59 crypts/mouse, and representative of 2 independent experiments. *, $P < 0.05$; **, $P < 0.01$, two-tailed unpaired Student's t test.

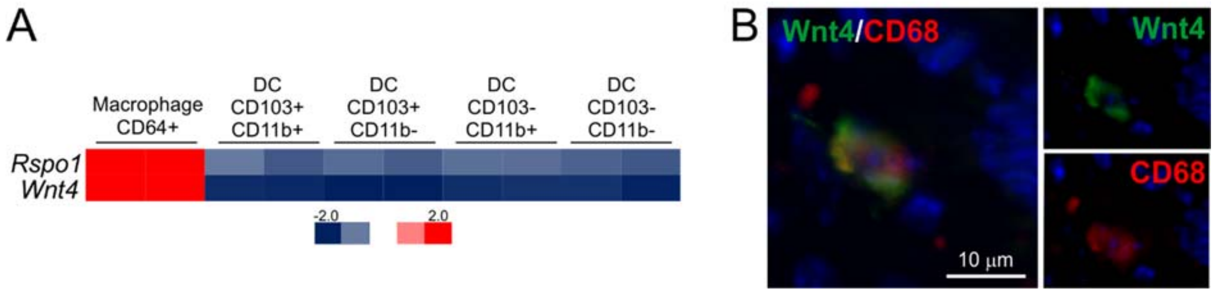

Supplementary Figure 9. (*A*) Heat map showing relative expression of *Rspo1* (upper row) and *Wnt4* (lower row) in microarray data from distinct mononuclear phagocyte populations isolated from the small intestine (GEO data set: GSE100393). DC, dendritic cells. (*B*) IHC analysis of Wnt4 expression (green) by CD68^{+} macrophages (red) in the gut wall. Sections were counterstained with DAPI to detect cell nuclei. Scale bar, 10 µm.

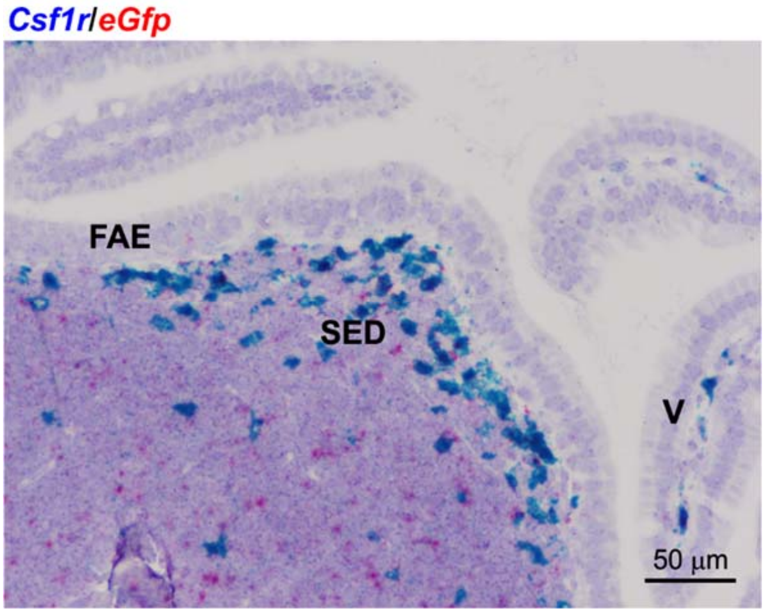

Supplementary Figure 10. Two colour mRNA *in situ* hybridisation analysis showed that the follicle-associated epithelium (FAE) overlying the Peyer's patch lacked expression of *Csf1r* (blue) and (red) *eGfp* mRNA. SED, subepithelial dome; V, villous. Scale bar, 50 µm.

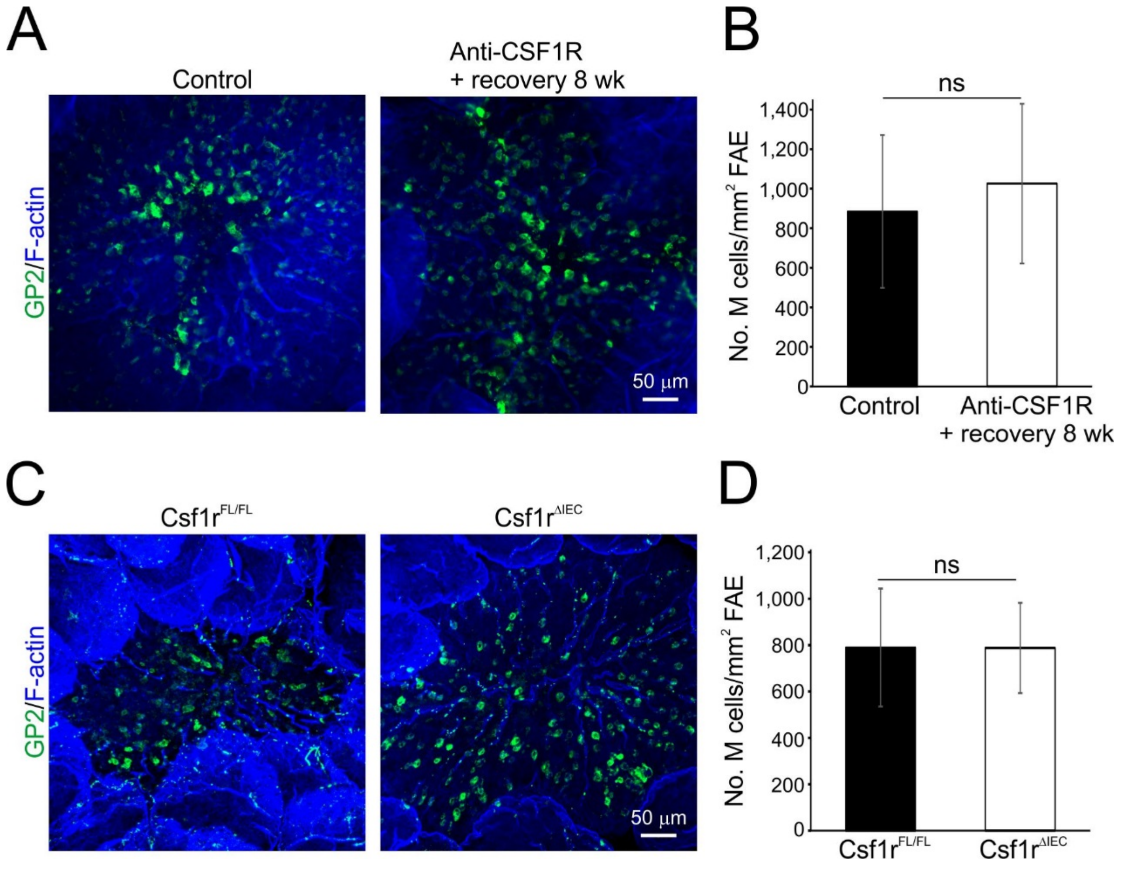

Supplementary Figure 11. (*A&B*) Mice were treated with anti-CSF1R mAb or control-IgG (control) for 6 weeks and then allowed to recover for 8 wk before analysis. (*A*) Peyer's patches were whole-mount immunostained to detect M cells ($GP2^+$ cells, green) and f-actin (blue). Scale bar, 50 µm. (*B*) Morphometric analysis showed that the density of $GP2^+$ M cells was restored within an 8 wk recovery period after prolonged CSF1R-blockade. Data were obtained from 2-4 mice/group, 2-3 Peyer's patches/mouse, with 2-3 follicle-associated epithelia analysed from each Peyer's patch. ns, two-tailed unpaired Student's *t* test. (*C*) Peyer's patches from $Csf1r^{FL/FL}$ mice (left-hand panel) and $Csf1r^{\Delta IEC}$ mice (right-hand panel) were whole-mount immunostained to detect M cells ($GP2^+$ cells, green) and f-actin (blue). Scale bar, 50 µm. (*D*) Morphometric analysis showed that similar densities of $GP2^+$ M cells were detected in the Peyer's patches $Csf1r^{FL/FL}$ mice and $Csf1r^{\Delta IEC}$ mice. Data were

obtained from 3 mice/group, 1-3 Peyer's patches/mouse with 4-10 follicle-associated epithelia analysed from each Peyer's patch. ns, two-tailed unpaired Student's *t* test.

Supplementary Table 1. Primary antibodies used for immunohistochemistry

**Target**	**Antibody details**	**Clone**	**Source**	**Dilution used**
Anxa5	Rabbit anti-annexin V	polyclonal	Abcam (Cambridge, UK)	1/400
CCL20	Rabbit anti-mouse CCL20	polyclonal	R&D Systems (Abingdon, UK)	1/200
CD11c	Hamster anti-mouse CD11c	HL3	BD Biosciences (Oxford, UK)	1/100
CD45R (B220)	Rat anti-mouse B220	RA3-6B2	Invitrogen (Paisley, UK)	1/100
CD68	Rat anti-mouse CD68	FA-11	Biolegend	1/100
GP2	Rat anti-mouse GP2	2F11-C3	MBL International, Woburn, MA)	1/250
Ki67	Rabbit anti-Ki67	polyclonal	Abcam	1/100
Lysozyme	Rabbit anti-lysozyme	polyclonal	Abcam	1/200
RANKL	Rat anti-mouse CD254	IK22/5	eBioscience (Hatfield, UK)	1/250
SpiB	Sheep anti-mouse SpiB	polyclonal	R&D Systems	1/500
SOX9	Rabbit anti-mouse SOX9	EPR14335-78	Abcam	7 µg/ml
Wnt4	Rabbit anti-Wnt4	polyclonal	Abcam	1.25 µg/ml

Supplementary Table 2. Primers used for RT-qPCR analysis

Gene	Forward primer	Reverse primer
Anxa5	TTTCCGTTGCACGGAGTTGT	TTTCCTGGCGCTGAGCATT
Bmi1	AATTAGTTCCAGGGCTTTTCAA	CTTCATCTGCAACCTCTCCTCTAT
Ccl9	TACTGCCCTCTCCTTCCTCA	TTGAAAGCCCATGTGAAACA
Cd68	GCAACTCGAGCATCATTCTTTCACC	GATGAGAGGCAGCAAGATGGAC
Csf1r	CCTGAAGGTGGCTGTGAAGATG	GCTCCCAGAAGGTTGACGATG
Emr1	ATGTGGGGCTTTTGGCTGCT	TGAGTCACTTTGAAGACATT
Gapdh	GATACTGCACAGACCCCTCCA	GCAGTTCCGGTCATTGAGGTA
Gp2	GATACTGCACAGACCCCTCCA	GCAGTTCCGGTCATTGAGGTA
Lgr5	GGGAGCGTTCACGGGCCTTC	GGTTGGCATCTAGGCGCAGGG
Lyz1	GAGACCGAAGCACCGACTATG	CGGTTTTGACATTGTGTTCGC
Lyz2	ATGGAATGGCTGGCTACTATGG	ACCAGTATCGGCTATTGATCTGA
Marcksl1	TTTTGCCCTCCTGTGGATTCT	CCACTAGGCACAGCACAAGAGA
Olfm4	AGTGACCTTGTGCCTGCC	CACGCCACCATGACTACA
Sgne1	ACGGTTAAAAATGGCCTCAAGG	AAGGACCCAGATGCTGAAGACC
SpiB	AGCGCATGACGTATCAGAAGC	GGAATCCTATACACGGCACAGG
Tnfs11	GAAGGCTCATGGTTGGATGTGG	GTGACTTTATGGGAACCCGATG
Tnfrsf11a	CTGCCTCTGGGAACGTGACTGG	GGCTGACATACACCACGATG
Tnfrsf11b	CACCTTGAAGGGCCTGATGT	TTTTGGGAAAGTGGGATGTTTT
Wnt3	TCCTCCTCGGCGCTGCTTCT	CCAGGGCCAGGGACCACCAA
Wnt3a	TCAGGGGTGATACCAAGACC	GGGACTGCAAATCTTCCTCA

Supplementary Table 3. RNAscope RNA *in situ* hybridisation probes used

**Gene**	**Probe target region**	**Supplier catalogue no.**
Bmi1	3096-3549	466021
Csf1r	241-1212	428191
Defa1	2-427	445751
eGfp	628-1352	400751
Emr1	85-1026	317961
Lgr5	2165-3082	312171

References

1. Middendorp S, Schneeberger K, Wiegerinck CL, et al. Adult stem cells in the small intestine are intrinsically programmed with their location-specific function. Stem Cells 2014;32:1083-1091.
2. Grun G, Lyubimova A, Kester L, et al. Single-cell messenger RNA sequencing reveals rare intestinal cell types. Nature 2015;525:251-255.
3. FANTOM Consortium and the RIKEN PMI and CLST (DGT), Forrest AR *et al*. A promoter-level mammalian expression atlas. Nature 2014;507:462-470.
