# Supplementary material for: The role of CSF1R-dependent macrophages in control of the intestinal stem-cell niche
Source: Nat Commun. 2018 Mar 28;9:1272. doi: 10.1038/s41467-018-03638-6 (PMC5871851; doi:10.1038/s41467-018-03638-6)
Supplement: Supplementary file 2 — Description of Additional Supplementary Files(PDF 82 kb) [file 41467_2018_3638_MOESM2_ESM.pdf]

Description of Additional Supplementary Files

File Name: Supplementary Movie 1
Description: Whole-mount IHC analysis of intestinal crypts in the intestines of untreated *Csf1r*-EGFP mice. *Csf1r*-EGFP, green; lysozyme, red; F-actin, blue.
